# Changes Within H3K4me3-Marked Histone Reveal Molecular Background of Neutrophil Functional Plasticity

**DOI:** 10.3389/fimmu.2022.906311

**Published:** 2022-06-10

**Authors:** Paweł Piatek, Magdalena Namiecinska, Natalia Lewkowicz, Małgorzata Kulińska-Michalska, Zbigniew Jabłonowski, Mariola Matysiak, Justyna Dulska, Sylwia Michlewska, Marek Wieczorek, Przemysław Lewkowicz

**Affiliations:** ^1^ Department of Immunogenetics, Medical University of Lodz, Lodz, Poland; ^2^ Department of Periodontology and Oral Mucosal Diseases, Medical University of Lodz, Lodz, Poland; ^3^ Department of Urology, Medical University of Lodz, Lodz, Poland; ^4^ Department of Neurology, Medical University of Lodz, Lodz, Poland; ^5^ Genomed SA, Warsaw, Poland; ^6^ Laboratory of Microscopic Imaging and Specialized Biological Techniques, Faculty of Biology and Environmental Protection, University of Lodz, Lodz, Poland; ^7^ Department of Neurobiology, Faculty of Biology and Environmental Protection, University of Lodz, Lodz, Poland

**Keywords:** innate immunity, neutrophils, H3K4me3-marked histone, ChIP-Seq, sepsis, NMOSD, periodontitis

## Abstract

Neutrophils are a heterogenous population capable of both antimicrobial functions and suppressor ones, however, no specific pattern of transcription factors controlling this plasticity has been identified. We observed rapid changes in the neutrophil status after stimulation with LPS, pre-activating concentration of TNF-α, or IL-10. Chromatin immunoprecipitation sequencing (ChIP-Seq) analysis of histone H3K4me3 allowed us to identify various transcriptional start sites (TSSs) associated with plasticity and heterogeneity of human neutrophils. Gene Ontology analysis demonstrated great variation within target genes responsible for neutrophil activation, cytokine production, apoptosis, histone remodelling as well as NF-κB transcription factor pathways. These data allowed us to assign specific target genes positioned by H3K4me3-marked histone with a different pattern of gene expression related to NF-κB pathways, apoptosis, and a specific profile of cytokines/chemokines/growth factors realised by neutrophils stimulated by LPS, IL-10, or TNF-α. We discovered IL-10-induced apoptotic neutrophils being transcriptionally active cells capable of switching the profile of cytokines/chemokines/growth factors desired in resolving inflammation *via* non-canonical NF-κB pathway with simultaneous inhibition of canonical NF-κB pathway. As apoptotic/suppressive neutrophils induced by IL-10 *via* positioning genes within H3K4me3-marked histone were transcriptionally active, newly described DNA binding sites can be considered as potential targets for immunotherapy.

**Graphical Abstract:**

H3K4me3 histone ChIP-Seq analysis reveals molecular drivers critical for switching neutrophils from their pro- to anti-inflammatory properties.

## Introduction

In the light of recent advances, neutrophils may play a role as antigen-presenting cells, suppressor cells, as well as cells supporting the regeneration of damages in the central nervous system (1–4). Many researchers highlight their active role in the resolution of inflammation to clear tissues from infiltrated leucocytes, leading to restoring tissue architecture and function (5–7). Our previous studies demonstrated that neutrophils can become IL-10-synthesizing suppressor cells upon contact with LPS-stimulated Treg lymphocytes or after direct stimulation by IL-10. As IL-10 induces synthesis of IL-10 in non-stimulated neutrophils, the process is amplified in a positive feedback manner initiating massive apoptosis. Activation of neutrophils with proinflammatory factors results both in their prolonged lifespan and inability to re-polarize into IL-10-synthesizing suppressive cells (8–10). Therefore, persistent pre-activation or activation of neutrophils accompany numerous chronic diseases such as rheumatoid arthritis, systemic lupus erythematosus, acute respiratory distress syndrome, atherosclerosis, and neuromyelitis optical spectrum disorders (NMOSD) (11, 12). Further, activation of neutrophils is observed in sepsis, where peripheral blood neutrophils are characterized by impaired migration to the infection focus and inadequate antimicrobial responses.

Neutrophils, as the fastest-reacting cells in response to pathogens with loosely arranged nuclear chromatin facilitating access of transcription factors to DNA in a relatively short time after stimulation, are supposed to be ‘ready’ for rapid initiation of gene expression related to immune response in the peripheral blood. Increasing evidence supports that neutrophils are transcriptionally active cells with the ability to adapt their genome adequately to the risk of infection (13). The blueprint transcriptional controls in mice are runt-related transcription factor 1 (RUNX1) and krueppel-like factor 6 (KLF6) which modulate neutrophil maturation, NF-κB RelB, IRF5, and activator protein 1 (AP-1) drives neutrophil effector responses, whereas regulatory factor X2 (2RFX2) and NF-κB RelB promote survival (14). The vast majority of inflammatory gene expression in human leucocytes is under the control of NF-κB protein complex. It is involved in the rapid engagement of critical factors for neutralizing pathogens, recruiting other immunocompetent cells as well as inducing mechanisms connected with resolving inflammation (15). The signal-mediated NF-κB activation can be classified into canonical and alternative (non-canonical) pathways. Proinflammatory cytokines or a wide variety of pathogen-associated molecular patterns (PAMPs) activate classical NF-κB response *via* engagement of NF-κB essential modulator (NEMO)-dependent pathway, whereas non-canonical pathway is NEMO-independent and primarily described as immune cell responses *via* TNF-receptor superfamily members. The third pathway of NF-κB activation is termed ‘atypical pathways’ and comprises cell response to stress factors: oxidative, genotoxic, and organelle stress (16). Although five members of the NF-κB family, which create 15 homo- or heterodimers, can be activated by a great variety of stimuli mediating different dimer formation, NF-κB subunits are also subjected to post-translational modifications (PTMs) which are important for activation and crosstalk with other signalling pathways (17). All NF-κB subunits contain a conserved N-terminal Rel homology domain (RHD) that shares affinity for the κB DNA sequence motif 5’-GGGPuNNPyPyCC-3 (Pu, purine; Py, pyrimidine). The complexity of this transcriptional regulation system is also augmented by the fact that different NF-κB dimers show different preferences for DNA-binding sequences as well as several sites of NF-κB can be methylated by histone-modifying enzymes (18–20). Lu and Sark showed that the p65 subunit was not associated with histone-modifying enzymes until it was activated, suggesting that this modification took place only after NF-κB disconnection from the IkB (21, 22). Other studies provided evidence that NF-κB methylation occurs only in the nucleus where it can bind to DNA (23).

The specific activation of immune cells is mediated by the modification of ‘chromatin landscapes’ enabling diversified access and activity of regulatory elements that guarantee their plasticity during inflammation (24). Recent studies demonstrated a strong positive correlation between histone H3 lysine K4 trimethylated (H3K4me3) and histone H3 lysine K27 acetylated (H3K27ac) in mast cells upon immunoglobulin E-mediated cross-linking of the IgEε receptors (25). Gene transcription is controlled by enhancers, which interplay with gene promoters as gene-distal regulatory elements increasing gene transcription, characterized by the presence of H3K27ac histones (26). Contrary to enhancers, transcriptional start sites (TSSs), are located mainly within H3K4me3 domains which are located after the first exon bounding TSSs (27). The level of this chromatin modification within TSSs reflects the amount of transcription and high mRNA concentration (28, 29). These data put forward the hypothesis that the process of neutrophil pre-activation, which prepares neutrophils to a more intense response to pathogens, can be associated with the H3K27ac, contrary to neutrophil direct stimulation which is characterized by high dynamic reaction, therefore probably is mediated by H3K4me3 positioning.

As we have previously shown, peripheral blood neutrophils are characterized by a high level of H3Ac and relatively low, but constant, H3K4me3. Disturbances within the positioning of H3Ac and H3K4me3 observed in neutrophils isolated from HIV individuals led to further abnormalities within H3K4me3 and its TSS gene regions. This phenomenon implicated LPS-mediated NF-κB canonical activation pathway and resulted from low amounts of κB DNA sites within histone H3K4me3-marked, low NF-κB RelA (p65), and TLR4 mRNA expression, as well as reduced free NF-κB RelA (p65) accumulation in the nucleus (30). These data emphasize an essential role of cooperation between H3K4me3-marked histone and NF-κB transcription factor in neutrophil functioning.

In this study, we used ChIP-Seq analysis of histone H3K4me3-marked, to identify various TSSs associated with LPS-, TNF-α-, and IL-10-stimulated neutrophils from healthy individuals, as well as neutrophils derived from patients with sepsis (systemic septic inflammation with LPS-stimulated neutrophils), NMOSD (aseptic inflammation with pre-activated neutrophils), and periodontitis (local self-limiting septic inflammation with IL-10-positive neutrophils). We provided comprehensive epigenomic analysis within H3K4me3-marked histone that allowed us to identify human neutrophil regulators affecting their plasticity during inflammation as well as suppression.

## Materials and Methods

### Study Design

This study aimed to define mechanisms involved in neutrophil plasticity during inflammation as well as suppression. As human neutrophils are characterized by a short lifespan (5-8h) and loosely arranged chromatin we assumed that rapid changes observed in neutrophils can be associated with direct access of transcription factors to TSSs. To achieve this goal, a comparative analysis of DNA binding to histone H3K4me3, as the richest in TSSs, was performed using ChIP-Seq methodology. The first step of investigations was performed on neutrophils isolated from the healthy volunteers under different stimuli, that mimic the natural process of their pre-activation, activation, or suppression. 2x10^6^ of healthy neutrophils were incubated *in vitro* without stimulation and in the presence of 100ng/ml ultrapure LPS from *E. coli* (serotype R515, Alexis Biochemicals) as the proinflammatory direct stimulator, 100ng/ml TNF-α as factor pre-activating neutrophils in a low concentration, and 100ng/ml IL-10 as factor polarizing neutrophils to immunosuppressive cells in RPMI 1640 for 5 h (5% CO_2_, 37°C, humid atmosphere).

In the second step, we performed validation of obtained results from the *in vitro* model by comparison to the clinical status where neutrophils were stimulated *in vivo*. The comparison included four groups: healthy people; patients with NMOSD (as neutrophils pre-activated by TNF-α); patients with periodontitis (as IL-10-stimulated neutrophils); and patients with sepsis caused by Gram-negative bacteria in the bloodstream (as LPS-stimulated neutrophils). Isolated neutrophils were incubated for 5h in RPMI under similar conditions as neutrophils used in the *in vitro* model.

There were four independent replicates for each experiment in the *in vitro* model. To validate results from ChIP-Seq analysis obtained from the *in vitro* model, three representative patients with functional test parameters closest to the mean values of their group were classified to ChIP-Seq analysis.

### Patients

#### NMOSD

Three patients with confirmed AQP4-IgG seropositive NMOSD, fulfilling the 2015 Wingerchuk criteria, were selected for ChIP-Seq analyses (31). All the patients were without systemic steroids or other anti-inflammatory drugs for at least 3 months before the study.

#### Sepsis

Six patients with sepsis, who developed at least two symptoms from the following criteria: body temperature above 38°C or below 36°C; pulse rate > 90/min; respiratory rate > 20/min or PaCO_2_ < 32 mmHg; leukocyte > 12000/µl or < 4000/µL were classified for investigations (32). Patients that had not been receiving antibiotics for at least 3 months before the study, and with positive microbial tests for *E.coli* in the blood, were selected for ChIP-Seq analyses.

#### Periodontitis

Twelve patients with generalized stage III and IV periodontitis were selected for this study. The criteria of the Classification of Periodontal and Peri-Implant Diseases and Conditions 2017 (33) were used for periodontitis staging and classified for further investigation. Patients that had not been receiving antibiotics or anti-inflammatory drugs for at least 3 months before the study were selected for ChIP-Seq analyses.

All participants of the study were diagnosed and recruited at the Department of Neurology (patients with NMOSD), the Department of Urology (patients with sepsis), and the Department of Periodontology and Oral Mucosal Diseases (patients with periodontitis and healthy controls), all from Medical University of Lodz.

The Ethics Committee of Medical University of Lodz approved all protocols and informed written consent was obtained from all participants (RNN/25/15/KE, Medical University of Lodz).

### Neutrophil Isolation

We collected 20 mL of whole blood on lithium heparin anticoagulant from sepsis, NMOSD, and periodontitis patients as well as healthy controls (HC). Neutrophils were purified by negative selection by microbeads, which allowed the removal of DCs, B cells, monocytes, macrophages, activated T cells, and activated NK cells (MACSxpress Whole Blood Neutrophil Isolation Kit, Miltenyi Biotec GmbH, Germany). Residual erythrocytes were lysed with the use of 2 mL ammonium chloride Lysing Reagent (BD Biosciences) for 5 min. The final purity of PMN population was assessed by flow cytometry using CD14-PE (clone M5E2), CD15-FITC (MMA), and CD16-PECy7 (3G8, all from BD Pharmingen, San Diego, CA, USA) mAbs. Flow cytometric analysis of the isolated population of cells showed that the percentage of CD15^high^CD16^+^CD14^-^ neutrophils was >98%. The level of contaminating CD14^+^CD15^+^ monocytes was about 0.4% and CD15^+^CD16^-^ eosinophils was <0.1% after isolation (**Figure S1A in Supplementary Material**).

### Phagocytosis and Reactive Oxygen Intermediate (ROI) Production

Ready-to-use kits were used for the analysis of neutrophil phagocytosis and ROI production (Bursttest and Phagoburst, OrphoGen Pharma, Germany). Measurements of peripheral blood neutrophil function were performed in the whole blood (experiment performed on patients: MNOSD, sepsis, or periodontitis) according to the manufacturer’s instruction, whereas experiments with neutrophils stimulated by LPS, TNF-α, or IL-10 were performed on isolated cells. Samples were analysed within 30 min after incubation with LPS, TNF-α, or IL-10 using flow cytometry (BD LSRII, FACSDiva™).

### CD11b/CD18, Expression

Adhesion molecule expression was measured on the surface of neutrophils isolated from the whole blood. Experiments were performed on two groups: patients with NMOSD, sepsis, and periodontitis, as well as on healthy controls with/without stimulation by LPS, TNF-α, or IL-10. 2x10^6^ cells/ml neutrophils suspended in PBS were incubated at RT with conjugated monoclonal antibodies: anti-CD11b-PE (ICRF44, BD), CD18-FITC (L130, BD). After 30 min of incubation and rinsing, the samples were fixed with 1% formaldehyde and analysed (LSRII, BD).

### Immunocytochemical Analysis (ICC)

ICC analysis was performed on isolated neutrophils (from healthy volunteers) stimulated by LPS, TNF-α, or IL-10 (*in vitro* model) and on neutrophils incubated in RPMI and obtained from patients: NMOSD, sepsis periodontitis (neutrophils stimulated *in vivo*). Neutrophils were transferred to gelatin-coated microscope slides by cytospin (300xg, 10 min) and fixed with 4% formaldehyde solution for 20 min at 21°C. Fixed cells were washed with PBS and blocked with 10% rabbit blocking serum (Santa Cruz Biotechnology, Dallas, TX, USA) supplemented with 3% TritonTM X-100 (Sigma-Aldrich, St. Louis, MO, USA) for 45 min at 21°C. Next, they were washed and double stained for NF-κB RelA/IκB, NF-κB2 (p100)/NF-κB RelB, H3K4me3/NF-κB RelA, AnnexinV/Caspase3, or NF-κB RelA/H3Ac. Anti-H3K4me3 (2 µg/ml, clone CMA304, mouse, Millipore, Temecula, USA), Anti-H3Ac (Lys4, rabbit Millipore, Temecula, CA, USA), anti-NFκB RelA(p65) (1:100, C-20, rabbit, Santa Cruz Biotechnology, Dallas, TX, USA), anti-NFκB RelA(p65) (1:100, 6D889, mouse, Santa Cruz Biotechnology, Dallas, TX, USA), anti-IκB (1:100, H4, mouse, Santa Cruz Biotechnology, Dallas, TX, USA), Annexin V (1:100, H-3, mouse, Santa Cruz Biotechnology, Dallas, TX, USA), anti-RelB (1:100, GeneTex, Alton Pkwy Irvine, CA USA), anti-NFκB2 (1:150, Proteintech, Rosemont, IL, USA), anti-Caspase 3 (2 µg/ml, 9H19L2, rabbit, Invitrogen, USA), and human IgG Isotype Control (Invitrogen, Cat.#31154) as negative primary antibody control, were used. All antibodies were suspended in PBS supplemented with 1.5% blocking rabbit serum, 0.3% Triton X-100, 0.01% sodium azide, and incubated overnight at 4°CC. Cells were washed, and secondary fluorescent Abs were added for 1h at RT: goat pAb to mouse TR (5 μg/ml, cat. T862, Invitrogen, USA) with goat pAbs to rabbit FITC (2 μg/ml, Invitrogen, USA) or goat pAb to mouse FITC (1:100, Abcam) with goat pAbs to rabbit TR (4 μg/ml, Invitrogen, USA). As isotype secondary antibody controls, goat IgG F(ab’)2 FITC (Invitrogen, Cat.#11301C) and rat IgG2a Texas Red (Invitrogen, cat# R2A17) were used. For nuclei DNA staining, DAPI (1.5 μg/ml UltraCruz Mounting Medium, Santa Cruz Biotechnology, Dallas, TX, USA) was used. The confocal laser scanning microscopy platform TCS SP8 (Leica Microsystems, Germany) with the objective 63×/1.40 (HC PL APO CS2, Leica Microsystems, Germany) was used for microscopic imaging. Leica Application Suite X (LAS X, Leica Microsystems, Germany) was used for cell imaging. Fluorescence intensity was determined in the Region of Interest as the sum of the fluorescence from all segments (bordered by the line) divided by their number (arbitrary units- a.u.). The average fluorescence was calculated using at least 100 single cells for each sample. Nonspecific fluorescence (signal noise) was electronically diminished to level when nonspecific signal was undetectable (background). ICC data were additionally presented as the values of overlap coefficient that indicate the overlap of the fluorescence signals between the channels FITC, TR, and DAPI (nucleus). It was calculated as the mean value from every single Region of Interest using Leica Microsystem (LAS – X, ver. 3.7.020979 software, Leica, Germany). The overlap coefficient ranges from 0 (no co-localization) to 1 (complete co-localization).

### Chromatin Immunoprecypitation (ChIP)

Neutrophils stimulated by LPS, TNF-α, or IL-10 (*in vitro* model) and isolated from patients: NMOSD, sepsis, periodontitis, and incubated in RPMI (neutrophils stimulated *in vivo*) were used to analyse DNA coupled with H3K4me3 histone. ChIP was carried out in neutrophils according to the manual of Magna ChIP™ A/G Chromatin Immunoprecipitation Kit (Merck Millipore). Cells were fixed with 1% formaldehyde in RPMI solution for 10 min. at RT, which was quenched with 10x glycine in 5-min incubation at RT to stop the fixation. After washing with cold PBS, cells were treated sequentially with 1x Protease Inhibitor Cocktail II, Lysis Buffer with Protease Inhibitor Cocktail II, and Protease Inhibitor Cocktail II with Nuclear Lysis Buffer. Supernatant was removed and the cell pellet was resuspended in Nuclear Lysis Buffer. Sonication (10 cycles; 30sec. “ON” 30sec. “OFF”) was done using Bioruptor^®^ Pico Sonicator (Diagenode, Belgium). The obtained chromatin was spun at a minimum of 10,000 x g at 4°C for 10 min to remove insoluble material. Each immunoprecipitation required the addition of Dilution Buffer and Protease Inhibitor Cocktail II. 25 μL of the diluted chromatin as ‘Input’ was saved at 4°C for further proceeding. Chromatin immunoprecipitation was performed with the use of the set of antibodies: Normal mouse IgG (negative control), anti-RNA Polymerase II (clone CTD4H8) as positive control, and anti-trimethyl-Histone H3 (Lys4) (MC315, Merck Millipore) mAbs. Both antibodies were recommended for the use in ChIP-Seq technique (34). Immunoprecipitation reactions were incubated overnight at 4°C with rotation. DNA was eluted and purified using spin columns. The DNA concentrations of obtained samples were measured by Qubit 4 Fluorometer (ThermoFisher Scientific, Waltham, MA, USA).

### Library Preparation and NGS Sequencing

Double-stranded DNA was generated from single-stranded fraction of ChIPed DNA using NEBNext^®^ Ultra™ II Non-Directional RNA Second Strand Synthesis Module (E6111S, New England Biolabs, South Hamilton, MA, USA). Reaction was carried out in the presence of random primers from NEBNext^®^ RNA First Strand Synthesis Module (E7525, New England Biolabs). Libraries for sequencing were prepared using NEBNext^®^ Ultra™ II DNA Library Prep Kit for Illumina^®^ (E7645L, New England Biolabs). Single-end sequencing with read length of 75 bases (SE75) was performed with NextSeq550 (Illumina) in order to obtain at least 20 million reads per sample that could be mapped to the human genome (35). ChIP-Seq library quality control analysis is presented in **Figure S1B in Supplementary Material**.

### Bioinformatic Methodology of the ChIP-Seq Analysis

In the first stage, the quality of the raw sequence reads was checked using the FASTQC software (version: 0.11.8). Next, all reads were subjected to the adapter and quality filtering [minimum quality (-q 25), minimum length (-m 15)] using the Cutadapt tool (version: 1.18) in NextSeq reads mode. Trimmed reads were aligned to the reference genome (GRCh38) using the Bowtie2 (version: 2.2.9) in the single-end mode. Duplicated reads were located and tagged using the Picard MarkDuplicates tool (version: 2.18.4). Reads with a low mapping quality score (MAPQ <10) were removed from downstream analysis with the Samtools software (version: 1.6). Protein binding site identification in the previously prepared BAM files was performed with the MACS2 (Model-based Analysis of ChIP-Seq) software (version: 2.1.0) in narrow peak mode (36). Subsequently, identified peaks were annotated using annotatePeaks.pl from Homer software (version: 4.11.1, hg38 annotation library). Additionally, a functional enrichment analysis for various categories (molecular function, biological process, cellular component, and pathways interaction) was executed (37). To find enriched motifs in ChIP-Seq peaks the findMotifsGenome.pl program from Homer software (version: 4.11.1) was used. The quantitative assessment of ChIP-Seq quality was checked by applying the ChIPQC package (version: 1.21.0) from R Bioconductor (version: 3.6.0) (**Figure S1C in Supplementary Material**). Differentially enriched sites between experimental conditions were identified using the DiffBind package (version: 2.12.0) from R Bioconductor (version: 3.6.0).

### Human Chemokine Multiple Profiling Assays

54 Chemokine/cytokine/growth factor concentrations in neutrophil culture supernatants were measured using Bio-Plex Pro™ Human Chemokine Assays and Bio-Plex Pro™ Human Cytokine 27-plex Assay (Bio-Rad Laboratories). Isolated neutrophils from both experimental models (stimulated by IL-10, LPS, or TNF-α as well as isolated from patients: NMOSD, sepsis, periodontitis) were incubated for 5h at 37°C, 5% CO_2_, in a humid atmosphere. Standards and samples were diluted (1:4) in sample diluent and transferred to the plate containing magnetic beads for 1h at RT. The plate was washed (3x) and detection antibody was added for 30 min on a shaker (850 rpm) at RT. After that, the plate was washed (3x) and streptavidin-PE solution was added for 10 min. Subsequently, the plate was washed (3x) and samples were re-suspended in 125 µL of assay buffer and analysed within 15 min. All samples were analysed at the same time in duplicate. All reagents and technology were provided by Bio-Rad Laboratories (Bio-Plex 200).

### Multiple Gene Profiling Microarray

Total RNA was extracted from HC neutrophils stimulated by IL-10, LPS, or TNF-α (*in vitro* model) and neutrophils isolated from adequate clinical status incubated in RPMI with a mirVana™ miRNA Kit (Thermo Fisher Scientific). After isolation, the RNA level and the purity analysis were performed by an Agilent small RNA Kit (2100 Bioanalyzer, Agilent 2100 expert software). One hundred forty-four gene expression was analysed using Human NF-κB Signaling Pathway RT2 Profiler PCR Array and NFκB Signaling Targets RT2 Profiler PCR Array (both Qiagen, UK). cDNA was amplified in the presence of specific primers (RefSeq accession numbers provided in **Table S5 in Supplementary Material**), coated in 96-well microtiter plates on a 7500 Real Time PCR System (Applied Biosystems) according to the following program: 95°C 10 min (activation of HotStart DNA polymerase); 50 cycles of (95°C, 15s; 60°C, 60s). We used RT2 Real-Time™ SYBR Green/PCR Master Mix (Qiagen, UK) that contains all reagents and buffers required for qRT-PCR. The mean expression levels of the following housekeeping genes were used for the normalization of the cDNA samples: hypoxanthine phosphoribosyltransferase 1, β-actin, and glyceraldehyde-3-phosphate dehydrogenase. Data from real-time PCR were calculated using the ΔΔCt method and the PCR Array Data Analysis Template v3.0 (Qiagen, UK).

### Statistics

Arithmetic means and standard deviations were calculated for all parameters. A statistical analysis of differences was performed using the one-way ANOVA test. Scheffe’s test was used for multiple comparisons as a *post hoc* test when statistical significances were identified in the ANOVA test. *P ≤* 0.05 was considered as the significant difference. The Person coefficient of correlation was used to verify the results obtained in the *in vitro* model (HC neutrophils stimulated by IL-10, LPS or TNF-α) to adequate clinical status (periodontitis, sepsis, or NMOSD patients).

## Results

### Neutrophils Stimulated by IL-10 Increased Expression of CD11b/CD18 and Synthesized Growth Factors/cytokines/chemokines Despite their Apoptotic Status

**Figure 1:**
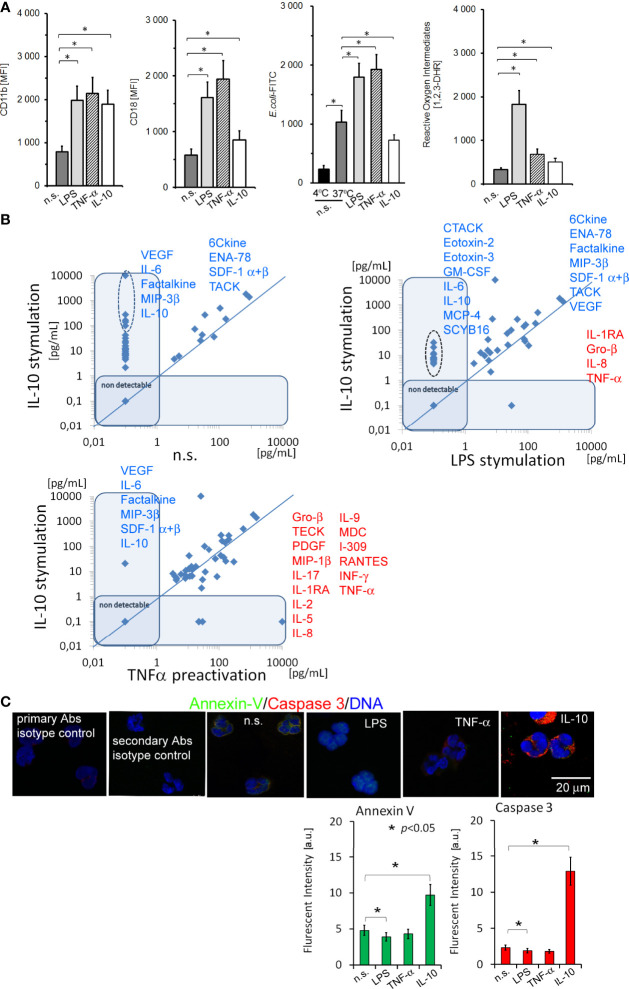
The analysis of adhesive molecule expression, phagocytosis, and ROI production, and the profile of cytokines/chemokines and growth factors released by neutrophils stimulated by IL-10 revealed their ability to synthesize a number of pleiotropic mediators, even though neutrophils were apoptotic. **(A)** The analysis of CD11b/CD18, *E.coli* phagocytosis, and ROI production by non-, LPS-, TNF-α-, or IL-10-stimulated neutrophils. The bars represent the MFI ± SD calculated from four independent experiments. **(B)** The comparison of cytokine/chemokine/growth factors profile released by neutrophils stimulated by IL-10 to non-stimulated and LPS or TNF-α−stimulated neutrophils. The blue line determines the border between up- and downregulated factors, each point represents the mean calculated from four independent experiments. **(C)** ICC double labelling for caspase-3 (red pseudocolor) and Annexin-V (green). The bars represent average fluorescence intensity ± SD calculated from four independent experiments, using at least 100 single cells for each test.

Neutrophils stimulated by 100ng/ml IL-10 for 5h increased CD11b adhesive molecule expression to the degree comparable with LPS or TNF-α stimulation, but contrary to proinflammatory stimuli, CD18 expression was significantly lower (**Figure 1A**). The ability of IL-10-treated neutrophils to phagocytosis or reactive oxygen intermediate (ROI) production was diminished (**Figure 1A**). Next, we found that IL-10 had a pleiotropic effect on the synthesis of different cytokines/chemokines and growth factors by neutrophils. Most spectacular changes exerted by IL-10 compared to no stimulation (n.s.) were observed in the concentrations of 6Ckine, CTACK, ENA-78, Eotoxin, -2, -3, Factalkine, G-CSF, GM-CSF, Gro-α, Gro-β, IL-2, IL-4, IL-6, IL-8, IL-10 (additive effect of exposition and synthesis), IP-10, MCP-4, MIF, MIP-1β,-1Δ, −3β, SCYB16, SDF-1α+β, TECK, and VEGF (**Figure 1B**). In addition, the comparison of neutrophils stimulated by IL-10 *vs.* LPS and TNF-α allowed us to identify highly specific mediators: VEGF, IL-6, Factalkine, MIP-3β, SDF-1 α+β, and IL-10, released by IL-10-stimulated neutrophils (**Figure 1B**). Neutrophils stimulated by LPS were characterized by more intensive synthesis of Gro-β, IL-1RA and IL-8 in comparison to IL-10 stimulation (**Table S1A in Supplementary Material**). We observed non-detectable levels of Eotoxin-2, -3, GCP-2, IL-6, IP-10, MCP-4, MDC, MIG, and SCYB16 after LPS stimulation but their higher concentrations after IL-10 or TNF-α stimulation. Neutrophils pre-activated by TNF-α were characterized by high production of Gro-α, I-309, IFN-γ, IL-1RA, -2, -5, -8, -9, -15, -17, MDC, MIP-1β, PDGF-bb, RANTES, TNF-α (additive effect of exposition and synthesis), and VEGF, but low production of ENA-78, IL-6, IL-10, MIP-3β, SDF-1α+β, and TECK in comparison to neutrophils stimulated by IL-10 (**Table S1A in Supplementary Material**). Finally, we showed that neutrophils stimulated by IL-10 possessed apoptotic markers assessed as Annexin V and caspase 3 expression, contrary to LPS- or TNF-α−stimulated neutrophils which were characterized by inhibition of apoptosis (**Figure 1C**).

### IL-10, LPS, and TNF-α Triggered Different NF-κB Pathways

**Figure 2:**
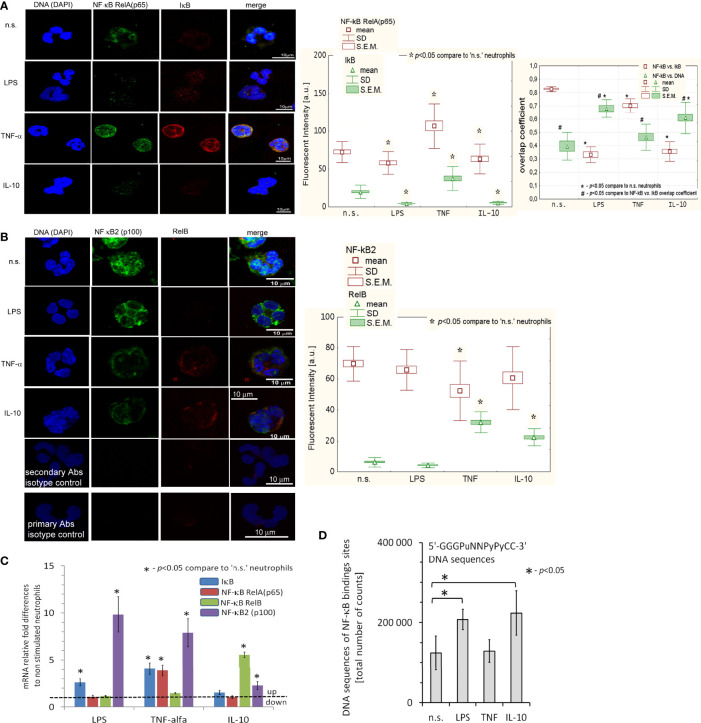
IL-10, LPS, and TNF-α triggered different NF-κB pathways. **(A)** ICC analysis displayed the neutralization of IκB junction with NF-κB RelA that initiated a migration of RelA (p65) unit to the cell nucleus. (Left panel) An example of ICC analysis. (Middle panel) Analysis of fluorescence intensity pointed to IκB and NF-κB RelA decrease in LPS- and IL-10-stimulated neutrophils contrary to TNF-α which amplified synthesis of IκB and NF-κB RelA as well as RelA translocation to the nucleus. The bars represent average fluorescence intensity ± SD and ± S.E.M. calculated from four independent experiments, using at least 100 single cells for each test. (Right panel) Overlap coefficient of NF-κB RelA *vs.* IκB and NF-κB RelA *vs.* DNA statistic comparison confirmed cleavage of NF-κB RelA bond with IκB after LPS, TNF-α and IL-10 stimulation of neutrophils which resulted in co-localization of RelA (p65) into the cell nucleus. The bars presented average overlap coefficient ± SD and ± S.E.M. calculated from four independent experiments. **(B)** An example of ICC of NF-κB2 and RelB analysis and statistical comparison of fluorescence intensity. The bars represent average fluorescence intensity ± SD and ± S.E.M. calculated from four independent experiments using at least 100 single cells for each test. **(C)** NF-κB RelA, RelB, NF-κB2, and IκB mRNA expression analysis revealed transcription of NF-κB RelA and their inhibitor (IκB) in neutrophils after exposition to TNF-α and LPS, but not to IL-10. In turn, IL-10-stimulated neutrophils initiated RelB mRNA transcription. The graph presents the average values ± SD calculated from four independent experiments. **(D)** NF-κB DNA binding sites within H3K4me3 in n.s., LPS-, TNF-α-, and IL-10-stimulated neutrophils. The graph presents the average values ± SD calculated from four independent experiments of 5’-GGGPuNNPyPyCC-3 DNA sequences of the total number of counts within H3K4me3-marked histone (Pu, purine; Py, pyrimidine).

Next, we focused on canonical and alternative (non-canonical) NF-κB pathways triggered during neutrophil activation. Using ICC, we found that treatment of neutrophils with IL-10 (similar to LPS, but opposite to TNF-α) caused activation of the NF-κB subunit RelA (p65) and its translocation from the cytoplasm to the nucleus (**Figure 2A**). Under the same conditions, neutrophils stimulated by IL-10 or pre-activating concentration of TNF-α upregulated expression of RelB. Another representative factor of non-canonical NF-κB pathway, NF-κB2 (p100), was not affected by IL-10 or LPS stimulation but was decreased after neutrophil pre-activation by TNF-α (**Figure 2B**). mRNA expression analysis revealed that, opposite to TNF-α or LPS, IL-10 stimulation did not result in the initiation of RelA or its inhibitor IκB, but RelB and NF-κB2 (p100) transcription (**Figure 2C** left panel) suggesting that IL-10-mediated process of neutrophil activation was exerted by mechanisms different from these involving classical proinflammatory mediators, or during neutrophil pre-activation. Therefore, in the neutrophils stimulated by IL-10, NF-κB RelA acted only as an initiating factor, while after LPS stimulation, its intense transcription reflected a direct response to pathogens. In turn, neutrophils pre-activated by TNF-α were characterized by the high concentration of NF-κB RelA and IκB, both at the protein and mRNA level, but co-localization of NF-κB RelA to the nucleus was slightly visible suggesting that NF-κB was not fully activated but only prepared to more effective pathogen responses.

Different response of neutrophils to IL-10, contrary to LPS or TNF-α stimulation, suggests some changes in functional genome organization at the level of post-translational histone modifications that could correspond with NF-κB activity. Using double-staining ICC, we noticed an increased level of histone H3K4me3 in neutrophils stimulated by IL-10 or LPS which simultaneously corresponded with high expression of NF-κB in the nucleus. This result may suggest that NF-κB was colocalized within H3K4me3-reach DNA regions (**Figure S2A in Supplementary Material**). NF-κB/H3K4me3-marked histone overlap coefficient was higher upon IL-10 stimulation (0.7 ± 0.04) *vs.* LPS (0.2 ± 0.02) pointing to direct initiation of TSS regions in IL-10-stimulated neutrophils (**Figure S2D**). In turn, neutrophils pre-activated by TNF-α were characterized by low H3K4me3 and high H3Ac levels as well as simultaneous translocation of NF-κB RelA to the cell nucleus (**Figure S2B, D**). Technical limitations caused by the resolution of the confocal fluorescence microscope, which is 17-23 times lower than the histone size, only allowed us to deduce about the regions of nuclei with NF-κB RelA and their location within the histone (**Figure S2E**). However, we could conclude the close relationship between NF-κB and H3K4me3-marked histone with the number of NF-κB binding sites within H3K4me3-marked histone.

Using ChIP-Seq technique, we noticed a large number of NF-κB binding sites within H3K4me3-marked histone in neutrophils stimulated by IL-10 or LPS, but not by TNF-α in comparison to n.s. neutrophils (**Figure 2D**), proving NF-κB and H3K4me3-marked histone crosstalk.

### DNA Annotation Within H3K4me3-marked Histone Revealed Changes in TSS Regions During Neutrophil Stimulation by IL-10 or LPS

**Figure 3:**
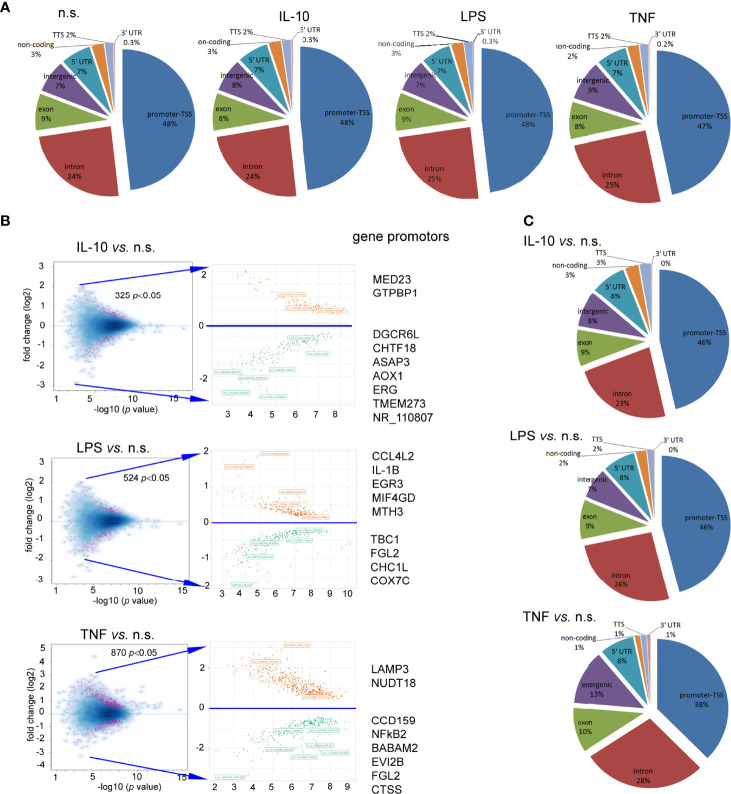
Neutrophils stimulated by IL-10, TNF-α, or LPS equally impacts each DNA annotation, indicating the involvement of various DNA components within H3K4me3-marked histone. **(A)** Distribution of the DNA annotation in H3K4me3-marked histone after stimulation by IL-10, LPS, or TNF-α. **(B)** Volcano plots demonstrated statistical significance in peak density compared to n.s. neutrophils from four independent biological replications. **(C)** Distribution of the DNA annotation within statically significant peaks associated with H3K4me3 revealed the most significant changes observed in the TSS region during the exposition of neutrophils to IL-10 or LPS. Each graph was performed based on the mean value calculated from four independent experiments; n.s, non-stimulated.

**Figure 4:**
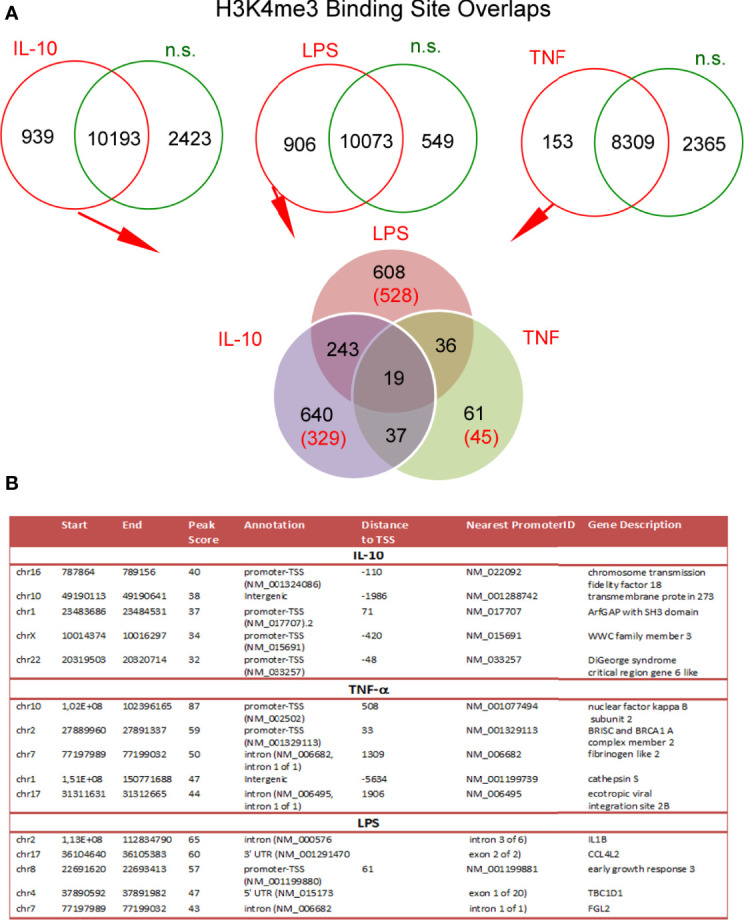
Binding site overlaps within H3K4me3-marked histone pointed DNA associated region with specific activation by IL-10, TNF-α, or LPS stimulation. **(A)** Pie chart of H3K4me3 binding site distribution in comparison to different stimuli. The number of statistically significant DNA binding sites with the low FDR is given in brackets. Pie charts were performed on the mean values obtained from four independent experiments. **(B)** Detailed characterization of IL-10, TNF-α, and LPS specific binding sites with the five highest statistical significances and the lowest FDR; n.s, non-stimulated.

To determine the changes within H3K4me3-marked histone of neutrophils stimulated by IL-10, LPS, or TNF-α in comparison to n.s. cells we used the ChiP-Seq technique. DNA annotation describing the function of detected DNA in H3K4me3-marked regions revealed only slight differences between the examined groups. The rates of TSSs were at 47%-48% regardless of stimuli (**Figure 3A**). Based on the computational algorithm described as model-based analysis of ChIP-Seq (MACS), we noted 325 differences in peak reading density for IL-10-, 524 for LPS-, and 570 for TNF-α−stimulated neutrophils in comparison to n.s. neutrophils within H3K4me3-marked histone (**Figure 3B**). All selected peaks were characterized by statistical significance and a very low empirical false discovery rate (FDR) value <0.01. The vast majority of differences were found within the TSS regions, especially in the neutrophils stimulated by IL-10 or LPS. Forty-eight percent of DNA annotation was assigned to TSSs in IL-10-treated neutrophils, 46% after LPS, and 38% after TNF-α (**Figure 3C**). Based on the MACS algorithm, all selected peaks have also been assigned to appropriate detailed annotation, nearest promoter ID, distance to TSSs as well as gene descriptions (**Figure 3B** right panel). We identified protein binding sites within histone H3K4me3-marked specific for the neutrophils stimulated by IL-10, but not associated with TNF-α pre-activation or LPS stimulation. Therefore, we performed a double-step analysis. First, we selected all specific H3K4me3 peak binding sites induced by differently stimulated neutrophils in comparison to n.s. neutrophils (**Figure 4A** upper panel). In the second step, the selected binding sites within H3K4me3 peaks specific for the certain stimulation were analysed to eliminate those that were mutual and independent of the stimulation type. Binding sites overlap analysis of allocating genes revealed 640 peaks (329 with low FDR) within H3K4me3-marked histone specific for IL-10 stimulation, 608 peaks (528 with low FDR) for LPS, and 61 peaks (45 with low FDR) for TNF-α (**Figure 4A** low panel). The example of the five peaks within H3K4me3 with the highest specificity for different stimuli is presented in **Figure 4B**. The description of all identified peaks, with the division of particular pie chart compartments, is attached in **Table S2 in Supplementary Material**.

### IL-10, LPS, or TNF-α Regulated H3K4me3-marked Histone Target Gene Profile in Neutrophils and Determined Their Plasticity

**Figure 5:**
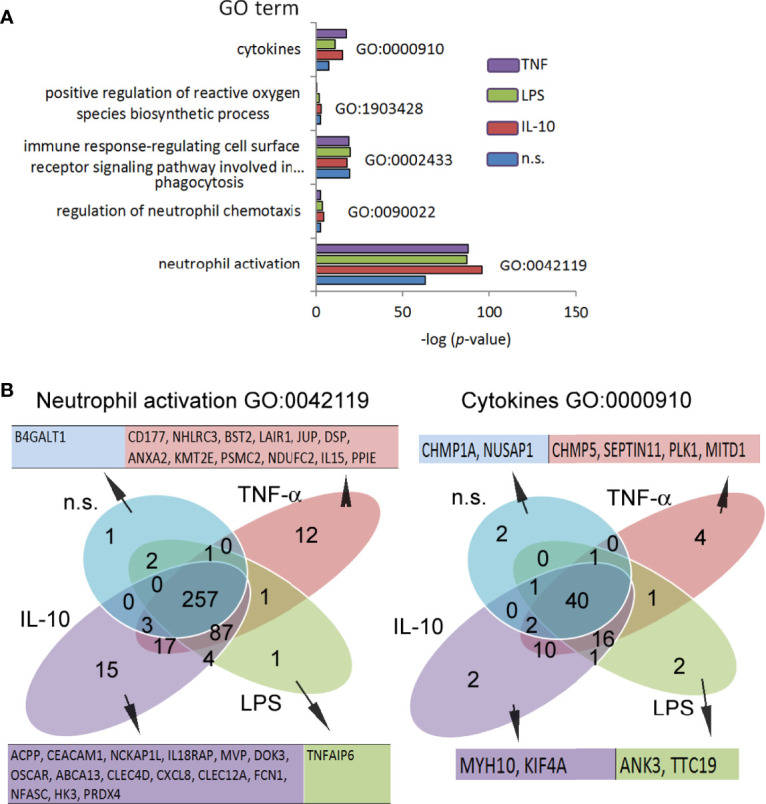
Neutrophils stimulated by IL-10, LPS, or TNF-α regulated H3K4me3-marked histone gene profile, determined different effects they exert during inflammation. **(A)** Gene Ontology analysis of the main processes responsible for neutrophil properties. Graph represents mean value calculated from four independent experiments. **(B)** Binding site overlap in the GO term ‘neutrophils activation’ and ‘cytokines’; n.s, non-stimulated.

Numerous changes in the TSS regions affect the metabolic processes of the cell. Consequently, we performed Gene Ontology (GO) analysis (37). GO components displayed many significant differences between investigated stimuli in comparison to the n.s. neutrophils (**Table S3 in Supplementary Material**). In particular, in IL-10-treated neutrophils, the following terms were upregulated within the processes with the highest differences in relation to n.s. neutrophils: ‘p53 pathway,’ ‘Class I PI3K signalling events,’ ‘IL1-mediated signalling events,’ ‘Alternative NF-κB pathway,’ ‘IL2-mediated signalling events’. In the neutrophils stimulated by LPS, the terms ‘p53 pathway’ and ‘regulation of telomerase’ were most significantly upregulated. In turn, stimulation of neutrophils by TNF-α activated ‘validated targets of C-MYC transcriptional activation’ and ‘p53 pathway’ terms. It is noteworthy that an increased density within H3K4me3-marked histone of the target genes in ‘mTOR signalling pathway’ dominated within all processes, regardless of stimuli (**Table S3 in Supplementary Material**). The analysis of all genes in this term revealed 41 target genes shared by n.s., IL-10-, and LPS-stimulated neutrophils, two genes (CCNE1 and CDK2) specific for both LPS and IL-10, and one gene (RRN3) specific for LPS stimulation. Neutrophils pre-activated by TNF-α shared 10 target genes with IL-10, LPS, and n.s. neutrophils (**Figure S3A in Supplementary Material**). The analysis of density reading within promotors of mTOR genes in H3K4me3-marked histone revealed its high expression during IL-10 and LPS, but not TNF-α stimulation (**Figure S3A** low panel). mTOR pathway and hypoxia-inducible factors (HIF) have been recognized as major regulators of metabolism in myeloid cells, which act together as a sensor of changes within the metabolic environment (38). We observed a high specific density of signals within gene promotors of HIF1α and HIF2α in IL-10-stimulated neutrophils, to a lesser extent in LPS-stimulated or n.s. neutrophils, but undetectable in TNF-α−stimulated neutrophils (**Figure S3B in Supplementary Material**). Next, we searched for the processes directly related to neutrophil activation and cytokine profile as the primary source of their diverse activities in inflammation. We found that neutrophils regardless of the stimulation type (IL-10, LPS, or TNF-α) were characterized by upregulation of the target genes within H3K4me3-marked histone in the terms: ‘neutrophil activation’ and ‘cytokines,’ but not in the terms ‘positive regulation of ROI biosynthetic process,’ ‘regulation of neutrophils chemotaxis,’ or ‘immune response-regulating cell surface receptor signalling pathway involved in phagocytosis’ (**Figure 5A**). As the major statistical differences were detected in the ‘neutrophil activation’ and ‘cytokines’ terms, with no differences regardless of the stimuli type, we focused on the particular target genes, and classified them into 15 groups based on the comparison of the peak density. We found that among 494 genes in the ‘neutrophil activation’ term, 15 target genes were highly specific for IL-10, 12 genes for TNF-α, 1 gene for LPS, and 1 gene for n.s. neutrophils. In the process of ‘cytokines,’ we noted only 2 specific target genes (MYH10, KIF4A) for IL-10, 4 genes for TNF-α (CHMP5, SEPTIN11, PLK1, MITD1), 2 genes for LPS (ANK3, TTC19) and 2 genes (CHMP1A, NUSAP1) for n.s. neutrophils (**Figure 5B**). The detailed list of target genes within specific subsets of neutrophils is presented in **Table S4A/B in Supplementary Material**.

### Changes within H3K4me3-marked Histone in Neutrophils Stimulated by IL-10 Affected Target Genes in Non-canonical NF-κB Pathway

**Figure 6:**
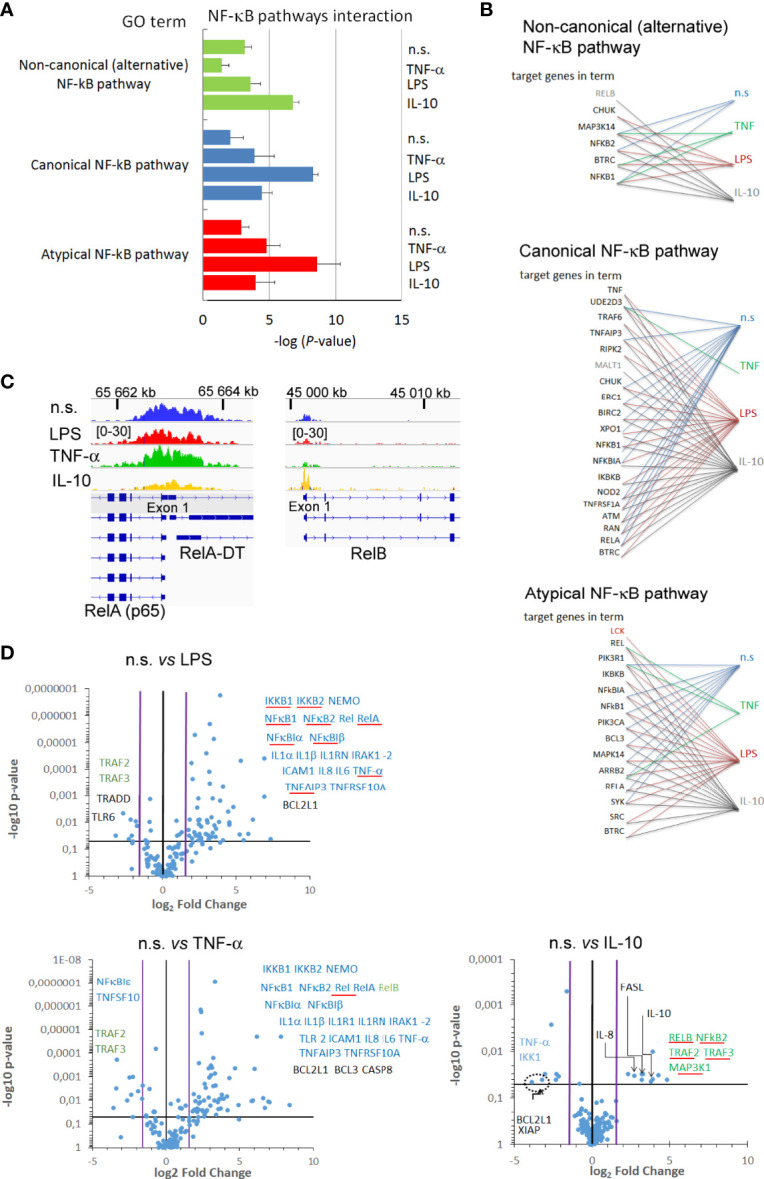
Changes within H3K4me3-marked histone in neutrophils stimulated by IL-10 affected target genes in non-canonical NF-κB pathway. **(A)** The analyses of GO terms: non-canonical, canonical, and atypical NF-κB pathways. The graph represents mean value ± SD calculated from four independent experiments. **(B)** The analysis of target genes in the GO terms NF-κB pathway within H3K4me3-marked histone. **(C)** The exemplary comparison of RelA, RelB, genes among different stimuli. **(D)** Different target genes proceeding in different stimuli directly reflected the profile of mRNA expression of pro- and anti-inflammatory factors. Volcano plot demonstrated statistical differences in value *vs*. relative fold changes to n.s. neutrophils. The blue horizontal line indicates the threshold for the *p*-value of the t-test (*p*<0.05); black vertical line indicates a fold-change in gene expression of 1; pink lines indicate the 3-fold-change in gene expression. Blue arrows indicate mRNA genes associated with canonical NF-κB pathway; green with non-canonical (alternative) and red atypical NF-κB pathway; circle dashed line bordered statistically significant pro-survival genes. Genes, in which mRNA expression corresponded with H3K4me3 GO analysis, are underlined with a red line; each point represents the mean calculated from four independent experiments; n.s, non-stimulated.

To determine the influence of H3K4me3-marked histone on NF-κB pathways, we performed Gene Ontology to analyse the processes engaged in NF-κB activity (**Figure 6**). Neutrophils stimulated by IL-10 involved target genes related to the alternative (non-canonical) and partially classical (canonical) pathways of NF-κB, in turn, LPS stimulation activated canonical and atypical NF-κB pathways. The pre-activating concentration of TNF-α upregulated target genes related to canonical and atypical NF-κB pathways within H3K4me3-marked histone, but not to alternative one (**Figure 6A**). Detailed analysis of target genes within specific pathways confirmed a strong correlation between individual genes and pathway activity (**Figure 6B**). Additionally, peak density overlap comparison confirmed GO analysis by visualization of high density within the peak assigned to the promotor region of NF-κB RelB (alternative NF-κB pathway) in the neutrophils stimulated by IL-10, while high-density peaks assigned to promotor region of NF-κB RelA (canonical pathway) were demonstrated in LPS-, TNF-α−stimulated, or n.s. neutrophils (**Figure 6C**).

Next, we considered how changes within H3K4me3-marked histone affected gene expression related to NF-κB activation and those regulating NF-κB activity. The analysis of peak density within gene promotors revealed 88 mRNA gene expressions among 144 examined that are positioned by H3K4me3 after IL-10 exposition, 81 after LPS, 7 after TNF-α stimulation, and 67 in non-stimulated neutrophils (‘*H3K4me3 TSS regions*’ column in **Table S5**). Among the above mentioned 88 genes, 10 genes were up- and 8 downregulated in IL-10-exposed neutrophils in comparison to n.s. cells (**Figure 6D**). Upregulated mRNA expression for NF-κB RelB, TRAF2, TRAF3, MAP3K14, and NFκB2, main factors of NF-κB non-canonical pathway, positively correlated with GO analysis. In turn, factors representing canonical NF-κB pathways such as IKK1 and TNF-α were decreased. In a similar comparison, LPS activation of neutrophils resulted in upregulation of 53 genes from 81 including typically assigned to the canonical NF-κB pathways. We noted a correlation between genes positioned by H3K4me3 and mRNA expression within the following factors related to the canonical NF−κB pathway: RelA, IKKB2, IKKB, TRAF6, TNFAIP3, CHUK, NF-κB1, NF-κBA, and TNF-α, and with the following factors related to atypical NF−κB pathway: LCK, REL, and BCL3. In turn, we noted downregulation of 11 genes including TRAF2 and TRAF3 in comparison to n.s. neutrophils which represent the non-canonical NF-kB pathway. Neutrophils pre-activated by TNF-α were also characterized by overexpression of genes related to canonical NF-κB pathways, but opposite to LPS-stimulation, neutrophils were additionally characterized by overexpression of BIRC3, CD27, CSF2, F8, IL1R1, IL2RA, NR4A2, RelB, TIMP1, TLR2, and TP53, and downregulation of CARD11, CXCL1, MAP3K1, NFκBIE, NOD1, RHOA, STAT1, and TICAM1-2 in comparison to n.s. neutrophils. Among all 57 upregulated genes in neutrophils pre-activated by TNF-α, we noted all NF-κB subunits, IKKB-1, 2, NEMO, and proinflammatory cytokines compared to n.s. neutrophils (**Figure 6D** and **Table S5 in Supplementary Material**). It is worth emphasizing that analysis of a correlation between genes positioned by H3K4me3 and mRNA expression within NF-κB pathways revealed that only Rel was dependent on H3K4me3, while the rest of the overexpressed genes were independent of H3K4me3 positioning in neutrophils pre-activated by TNF-α. This set of experiments signifies H3K4me3-marked histone positioning as the main phenomenon orchestrating the type of NF-κB pathway engaged after IL-10 and LPS stimulation of neutrophil, but not during pre-activation by TNF-α.

Since we showed that neutrophils stimulated by IL-10 became apoptotic, with a series of morphological changes including nuclear chromatin reorganization, the last set of experiments was focused on the GO terms associated with apoptosis within H3K4me3-marked histone. Neutrophil stimulation by LPS, TNF-α, or IL-10 resulted in upregulation of ‘apoptotic process,’ but had no effect on ‘regulation of execution phase of apoptosis’ or ‘caspase binding’ (**Figure S4A in Supplementary Material**). The analysis in the term ‘neutrophil apoptotic process’ revealed its upregulation in IL-10-stimulated neutrophils, but its downregulation in LPS- or TNF-α−stimulated neutrophils. The more detailed analysis in this term pointed to Annexin 1, CD44, PIK3CB, and PIK3CD as target genes responsible for IL-10-induced apoptosis ‘positioned’ by H3K4me3-marked histone (**Figure S4B** right panel). In turn, analysis of the 568 target genes driving apoptosis in the term ‘apoptotic process’ revealed 19 DNA peak regions which were characteristic for IL-10, 24 peak regions for TNF-α, 12 peak regions for LPS, and 5 for n.s. neutrophils (**Figure S4B**). The detailed list of target genes within ‘apoptotic process’ in specific subsets of neutrophils is presented in **Table S4B in Supplementary Material**. As we did not observe any differences within ‘regulation of execution phase of apoptosis’ or ‘caspase binding’ processes, we focused on the factors involved in chromatin condensation, caspase-independent apoptosis pathway, and Bcl-2 family of apoptosis regulator proteins. H1 proteins, as linker histones, are associated with the condensation of nucleosome chains into higher-order structured fibres influencing nucleus morphology and gene expression (39). Reading density comparison of peak regions in differently stimulated neutrophils showed the highest density in regions within histone H1-0, HIST1H1C, HIST1H1D, and HIST1H1E proteins in IL-10-stimulated neutrophils (**Figure S4C in Supplementary Material**). Conversely, undetectable readings within BCL-2 or XIAP genes in H3K4me3-marked histone were found in neutrophils stimulated by IL-10, but not by LPS or TNF-α (**Figure S4D in Supplementary Material**). Moreover, no differences in the initiator caspase gene within H3K4me3-marked histone were found in the experimental setups. Since in transcription-active cell changes in nucleus morphology can be associated with poly(ADP-ribose) polymerase-1 (PARP-1), which is not only involved in DNA repair but also in caspase–independent apoptosis (40), we analysed PARP-1 in differently activated neutrophils and found the highest peak density within gene promotor of PARP-1 in neutrophils stimulated by IL-10 (**Figure S4E in Supplementary Material**).

### Changes within H3K4me3-marked Histone Positioned PDL-1L in Neutrophils Stimulated by IL-10

Recently, many researchers have postulated the hypothesis that circulating neutrophils are not a homogeneous population (41). Therefore, we speculated that markers, usually considered as immunophenotyping neutrophils into pro- *vs.* anti-inflammatory cells, were ‘positioned’ by H3K4me3-marked histone. Thus, we checked peak regions of CD177, glycoprotein olfactomedin-4 (OLFA4) (both typical for proinflammatory neutrophils), and PDL-1L (typical for immunosuppressive neutrophils). The comparison of the peak region reading density within CD177 or OLFA4 revealed no specific signalling regardless of the stimuli used. In turn, PDL-1L gene analysis revealed high reading density within gene promotors in neutrophils stimulated by IL-10 in comparison to n.s., TNF-α− or LPS-stimulated neutrophils (**Figure S5 in Supplementary Material**).

### Validation of *In Vitro* Data to Clinical Status

Finally, we have verified the obtained data by their extrapolation to adequate clinical status, using neutrophils derived from patients with sepsis (systemic septic inflammation with LPS-stimulated neutrophils), NMOSD (aseptic inflammation with pre-activated neutrophils), and periodontitis (local self-limiting septic inflammation with IL-10-positive neutrophils). Correlation analysis between *in vitro* and *ex vivo* profiles of cytokines confirmed correspondence between healthy neutrophils stimulated *in vitro* by LPS with sepsis neutrophils (R^2 ^= 0.83), TNF-α−pre-activated neutrophils with NMOSD neutrophils (R^2 ^= 0.67), and IL-10-stimulated neutrophils with periodontitis neutrophils (R^2 ^= 0.77) (**Figure S6A** and **Table S1A vs. S1B in Supplementary Material**). Next, we analysed the correlation for NF-κB-associated gene expression which also confirmed correspondence between LPS-stimulated neutrophils with sepsis neutrophils (R^2 ^= 0.83), TNF-α−pre-activted neutrophils with NMOSD neutrophils (R^2 ^= 0.75), and IL-10-stimulated neutrophils with periodontitis neutrophils (R^2 ^= 0.98) (**Figure S6B** left panel in Supplementary Material). Analysis of gene expression demonstrated that only a few genes were statistically differently expressed by *in vitro* stimulated neutrophils *vs.* respective *ex vivo* neutrophils; confirming that the clinically matched diseases were adequately selected (**Figure S6B** right panel in Supplementary Material). Consequently, the ICC microscopy of RelA/H3K4me3, as well as H3Ac levels in *ex vivo* neutrophils, corresponded with the *in vitro* model (**Figure S6C in Supplementary Material**).

**Figure 7:**
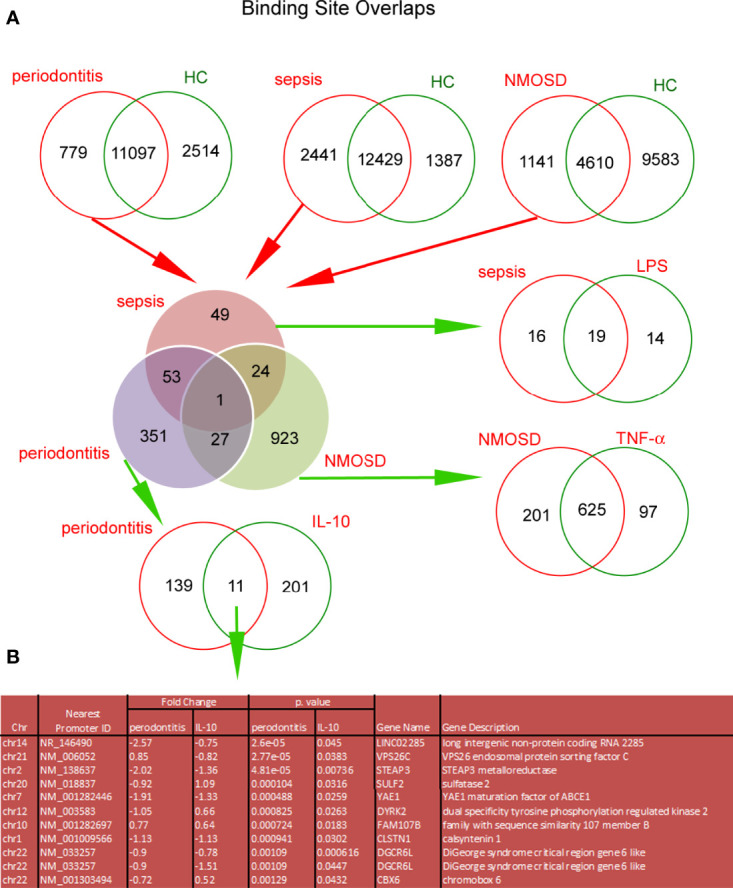
The comparison of Binding Site Overlaps between *in vitro* model of neutrophil polarization and adequate clinic status. DNA binding sites within H3K4me3-marked histone in freshly isolated neutrophils from patients with sepsis, NMOSD, and periodontitis were juxtaposed with DNA binding sites associated with specific activation in healthy volunteer neutrophils stimulated by LPS, TNF-α, or IL-10 for 5h, respectively. **(A)** The first stage of comparison has indicated unique sites for each clinical condition. In the next step, the specific binding sites have been narrowed down to those that occur selectively only in sepsis, NMOSD, or periodontitis - elimination of binding sites from each group as the result of neutrophil non-specific activation regardless of the immune circumstances. In the last step of the analysis, the unique binding sites were compared with data obtained from *in vitro* model. Binding Site Overlaps graph was performed based on the average value of *in vitro* model (four independent experiments) and the average value of two independent adequate clinical cases. **(B)** Detailed summary table of high specificity binding sites with the highest statistical significance and the lowest FDR peaks which characterized polarization of neutrophils into immunosuppressive cells.

Similar to the *in vitro* model, H3K4me3-marked histone ChiP-Seq analysis revealed a large variation in DNA binding sites within H3K4me3-marked histone between sepsis, NMOSD, and periodontitis neutrophils (**Figure S7A in Supplementary Material**). We juxtaposed all the DNA binding sites within H3K4me3-marked histone unique for each clinical type of neutrophils with the unique sites for the *in vitro* model. We noted 11 common DNA binding sites in periodontitis and IL-10-stimulated neutrophils, 625 in NMOSD and TNF-α-stimulated neutrophils, and 19 in sepsis and LPS-stimulated neutrophils (**Figure 7A**). **Figure 7B** presents 11 highly specific binding sites characteristic for neutrophils stimulated by IL-10 and periodontitis neutrophils within H3K4me3-marked histone. Further bioinformatics analysis allowed us to identify the following peaks nearest to gene promoter: LINCO2285, VPS26C, STEAP3, SULF2, YAE1, DYRK2, FAM107B, CLSTN1, DGCR6L, and CBX6.

Even though DNA binding site examination showed only a little correspondence between *in vitro* models and respective diseases, GO processes that emerged in *in vitro* experiments were also activated in the neutrophils isolated from the patients. Periodontitis, sepsis, and NMOSD neutrophils were characterized by target genes within H3K4me3 that positively controlled ‘neutrophils activity’ and ‘cytokine production’ processes. The non-canonical NF-κB pathway dominated in the periodontitis neutrophils, canonical NF-κB pathway in sepsis, NMOSD, and periodontitis neutrophils, while atypical only in sepsis neutrophils (**Figure 6B**; **Table S3 in Supplementary Material**).

Results of ChiP-Seq studies are available in the repository on the NCBI PubMed website (GEO https://www.ncbi.nlm.nih.gov/geo/query/acc.cgi?acc=GSE186508).

## Discussion

In this study, we demonstrated that neutrophils can change their status from pro- to anti-inflammatory cells in a relatively short period of time, and identified regulators within H3K4me3-marked histone responsible for their functional plasticity. The process of neutrophil polarization by IL-10 results in triggering a number of factors required not only to reduce the inflammation process (e.g., TNF-α synthesis) but also desirable to start the tissue renewal by chemokines recruiting macrophages and releasing growth factors critical *inter alia* to angiogenesis. As we have proven, these features are mainly orchestrated by epigenetic modification of histone H3 tri-methylation at the fourth lysine, which level is enhanced during neutrophils exposition to IL-10.

First, we discovered that neutrophils stimulated by IL-10 possessed the ability to synthesize and release a number of cytokines/chemokines and growth factors and increase expression of CD11b/CD18, despite being apoptotic. Among 54 examined factors released by IL-10-stimulated neutrophils, our attention was caught by the high concentration of vascular endothelial growth factor (VEGF) and CXC chemokines which might be the candidate factors critical for the resolution of inflammation. VEGF is considered as the main factor for endothelial and epithelial repair (42), while CXC chemokines are commonly regarded as mediators recruiting and moderating the function of macrophages and dendritic cells (43). The profile of CXC chemokines associated with induction of angiogenesis by ligation of CXCR2 receptor was represented in our study by CXCL1, CXCL2, CXCL5, CXCL8, CXCL10, and MIF as well as CCL19. Moreover, VEGF exerts its multidirectional action by direct stimulation of extracellular matrix remodelling, enhancement of endothelial cell proliferation and their migration, and increasing vascular permeability that results in the leakage of fibrinogen into the perivascular environment. Although neutrophil-derived VEGF was previously described, its role in wound healing has not been determined. Recently, proangiogenic CXCR4^hi^VEGFR^+^CD49d^+^ subpopulation of neutrophils recruited by VEGF-A following injury has been described (44). Similar to IL-10-stimulated cells, neutrophils stimulated by LPS or primed by TNF-α *in vitro*, as well as from the patients with sepsis or NMOSD, also released VEGF, but with a different pattern of chemokines (IL-8, CCL4, CCL5) and cytokines (IL-2, -5, -9, -15, -17, TNF-α) promoting inflammation. These data prompted us to postulate that neutrophils take an active role in initiating tissue healing after infection. The process is mediated by macrophages supporting and activating stem/progenitor cells, clearing damaged tissue, remodelling extracellular matrix to prepare scaffolding for regeneration, and initiation of angiogenesis. The role of IL-10 in the resolution of inflammation was further supported by our findings in neutrophils isolated from the patient with periodontitis. We noted a similar pattern of mediators released by non-stimulated periodontitis neutrophils and their gene expression, and neutrophils isolated from healthy volunteers stimulated *in vitro* by IL-10.

Neutrophils leaving the bone marrow are prepared for rapid polarization into effector antimicrobial or suppressor cells initiating resolution of inflammation. This might be possible due to nucleus morphology of neutrophils with loosely arranged chromatin (the lowest density among all leukocytes) that allows easier access of NF-κB to binding sites. In a previous study, we have demonstrated that euchromatin of resting neutrophils from heathy volunteers was characterized by high H3Ac acetylation and relatively low but constant H3K4me3 levels (30). In this study, we have noted a high level of H3K4me3-marked histone which corresponded with NF-κB RelA subunit activation in IL-10- and LPS-stimulated neutrophils. On the contrary, neutrophils pre-activated by low concentration of TNF-α were characterized by a low level of H3K4me3-marked histone with simultaneous high H3 acetylation and activation of canonical and atypical NF-κB pathways. The synergic changes within PTMs of H3 and NF-κB cell activation pathway were accompanied by the different response of neutrophils to LPS, IL-10, or TNF-α, affecting cytokine/chemokine/growth factor synthesis, ROI synthesis, *E.coli* phagocytosis, CD11b/CD18 expression, as well as apoptosis. Therefore, PTMs of H3 seems to be a primary process responsible for the plasticity and heterogeneity of circulating neutrophils. Our previous research as well as the current one suggests that mutual proportions of methylation *vs*. acetylation within H3, as well as the time of particular modification, determine the functional status of neutrophils according to the role they play at different phases of inflammation. This theory fits the general concept described by the Kouzarides group as histone modification ‘cross-talk’ which is an essential mechanism in coordinated cell plasticity (45).

In this study, we demonstrated the different functional outcomes of NF-κB RelA subunit activation by IL-10- and LPS. NF-κB RelA was activated and translocated to the nucleus after both LPS and IL-10 stimulation of neutrophils, however, contrary to LPS-stimulation, IL-10 did not induce RelA mRNA expression. This suggests only transient NF-κB RelA involvement in IL-10-treated neutrophils and inability to use NF-κB RelA later, thus, making the antimicrobial response of neutrophils *via* canonical NF-κB pathway impossible.

Subsequently, we performed Gene Ontology analysis of H3K4me3 and revealed that neutrophils stimulated by IL-10, opposite to LPS, engaged non-canonical NF-κB pathway genes identified as a pathway activated by TNF superfamily member receptor. This conclusion was supported by RelB and NF-κB2 (p100) expression both at protein and mRNA levels. Therefore, NF-κB RelA induced by IL-10, opposite to LPS or TNF-α stimulation, acts as a triggering factor switching neutrophils from proinflammatory activity that is dependent on NF-κB canonical pathway to non-canonical NF-κB pathway. The main difference between both NF-κB pathways is that canonical pathway-dependent activation is rapid and independent of protein synthesis whereas the non-canonical is slow and protein-dependent (46). In our opinion, it is physiologically justified as rapid activation is essential during infection, while protein synthesis requiring much more time is important during tissue regeneration.

Another unsolved aspect refers to the apoptosis process which coexists with the ability to synthesize factors promoting the reduction of inflammation as well as tissue renewal by neutrophils exposed to IL-10. Although NF-κB subunit RelA is known for its ability to inhibit apoptosis, it should be noted that it can be pro-apoptotic in response to certain stimuli that engage alternative NF-κB pathway genes (47). Cell treatment with atypical activators of NF-κB such as UV-C radiation or the chemotherapeutic drugs (daunorubicin and doxorubicin), switched RelA from a transcriptional activator into a gene-specific transcriptional repressor of antiapoptotic genes: BCL-xL, X-linked inhibitor of apoptosis protein (XIAP), and A20, but not *via* binding RelA-IκBα. This process resulted in cell apoptosis without the engagement of caspase activators (48). In turn, the study carried out on an immortalized mouse embryonic fibroblast (MEF) line demonstrated that constant exposition to H_2_O_2_ induced a caspase-independent, but PARP-1-dependent, cell death resulting in RelA activation mediating transcriptional repression of pro-survival genes BCL-xL or XIAP (49). In our study, we have noted that neutrophils stimulated by IL-10 were characterized by slightly increased ROI production, localization of RelA within the nucleus which corresponded with a high level of H3K4me3-marked histone, high rates of apoptosis, and engagement of non-canonical NF-κB pathway target genes within H3K4me3-marked histone. Therefore, we speculate that a model of apoptosis, similar to that described above by others, may be applicable to IL-10-stimulated neutrophils. In addition, in our experiment, the analysis of H3K4me3-marked peak density within PARP-1, BCL-2, and XIAP genes, considered critical for apoptosis induction *via* H_2_O_2_, revealed high readings within the transcription start site of PARP-1 but undetectable peaks within promotor for BCL-2 and XIAP in neutrophils stimulated by IL-10. The second evidence is provided by the analysis of gene expression associated with apoptosis. We have noted mRNA downregulation of BCL-2 and XIAP in neutrophils stimulated by IL-10 in comparison to n.s. cells. As a previous study demonstrated that induction of apoptosis *via* NF-κB was independent of initiator caspases (49), we checked expression for caspase-8,9,10 and found no significant changes in caspase 8 mRNA expression or peak density within caspases-8, -9 and -10 in the H3K4me3-marked histone. To conclude, participation of RelA-NF-κB and gene positioning within H3K4me3-marked histone directly affects apoptotic gene expression in IL-10-stimulated neutrophils.

Since the initial step of NF-κB RelA activation and its interaction with DNA after LPS or IL-10 stimulation seem alike, and the number of NF-κB binding sites was similar for both stimuli (**Figure 2E**), the next question is about alterations within NF-κB during IL-10 *vs.* LPS stimulation. In our opinion, the different functional outcomes might be a result of PTM processes that control NF-κB RelA subunit and specify target gene promotors. Among known PTMs, Thr505 phosphorylation has been shown to mediate pro-apoptotic effects upon cisplatin-induced DNA damage, and inhibition of NF-κB RelA target gene expression including anti-apoptotic Bcl-xL gene (50).

Noteworthy is the fact that all the observed changes in neutrophils take place in a relatively short time, suggesting that these cells are initially ready for three types of reactions: neutralizing pathogens, spontaneous apoptosis in the absence of infection, or initiating the tissue reconstruction after inflammation. The concept that neutrophils entering the periphery have nuclear chromatin prepared for rapid activation can be supported by several independent observations. First, relatively little rearrangement within DNA annotation of H3K4me3-marked histone after stimulation with LPS, TNF-α, or IL-10 in comparison to n.s neutrophils suggests that regardless of the nature of the signal (pro- or anti-inflammatory), neutrophil chromatin is prepared to initiate appropriate genes. Second, entering peripheral blood, neutrophils are also prepared for the inflammatory conditions associated with a low oxygen and glucose metabolism (51). Under physiological conditions, circulating neutrophils encounter a wide range of oxygen tensions, from13 kPa pO_2_ observed in the main arteries to 3 kPa in capillaries and veins (52). As hypoxia is significantly amplified in infected tissue, during ischemia due to damaged vasculature, neutrophils must be prepared for such extreme conditions (53). HIF1-α, together with HIF2-α and its inhibiting factor, are the sensitive sensors of low pO_2_, while mTOR-dependent cell signals act as sensors of environmental changes orchestrating cell metabolism (54). We revealed the engagement of mTOR target genes within H3K4me3-marked histone in neutrophils isolated from healthy individuals and stimulated *in vitro* with LPS or IL-10, as well as in neutrophils isolated from sepsis and periodontitis patients. Engagement of similar adaptive mechanisms to hypoxia in neutrophils stimulated by LPS and IL-10 could be explained by their activity in the site of inflammation, in contrast to pre-activating concentrations of TNF-α that mainly act in the peripheral blood. In sepsis, when peripheral blood neutrophils are pathologically activated by LPS, H3K4me3-marked histone was overexpressed and mTOR dominated within H3K4me3-marked histone. Positioning of HIF1α and HIF2α within H4K4me3 histone in non-stimulated, *in vitro* IL-10- or LPS-stimulated neutrophils, as well as in neutrophils isolated from the patients with corresponding clinical status, additionally confirmed that neutrophils entering blood vessels are already prepared for the inflammatory conditions. Further studies of HIF1α, HIF2α, and mTOR at mRNA and protein levels are necessary to verify this concept.

One of the important aspects of a hypoxic environment points to differences in metabolic processes which lead to apoptosis after IL-10 stimulation, and to inhibition of apoptosis after LPS stimulation. Walmsley et al. demonstrated that in a hypoxic environment, neutrophil apoptosis was inhibited by NF-κB RelA activation *via* HIF1-dependent response (51). They revealed that depletion of HIF1α in murine neutrophils resulted in a reduction of NF-κB RelA and IκB transcripts as well as anoxia-stimulated cell death. We noted that IL-10 caused NF-κB RelA translocation to trigger RelB and NF-κB2 (p100) synthesis in neutrophils and induction of non-canonical NF-κB pathway, whereas LPS amplified RelA synthesis and induced canonical NF-κB pathway. Likewise, neutrophil stimulation by IL-10 did not affect IkB mRNA expression, while LPS stimulation resulted in a significant increase in IkB mRNA.

The high positioning of processes within H3K4me3 that allow for rapid adaptation of neutrophils in response to a changing immune-environment might also explain the existence of different neutrophil populations in the peripheral blood. In the last decade, several studies highlighted the heterogeneity of neutrophils starting from their state of maturation, altered lifespan, ability to release cytokines like IL-10 or IL-17A, antigen presentation, diverse surface proteins, distinct antibacterial responses, proangiogenic, or immunosuppressive nature (41, 55). We confronted our data obtained from H3K4me3-marked histone analysis with previous findings suggesting heterogeneity of neutrophils based on the surface molecules. It was reported that in healthy individuals approximately 45%-65% of neutrophils were positive for CD177 and about 5%-40% for OLMA4, both representing antimicrobial activity (56–58). We did not observe characteristic peaks within CD177 and OLMA4 genes in H3K4me3-marked histone after IL-10, LPS, or TNF-α stimulation, thus the activity of these genes is independent of H3K4me3-marked histone. It is possible that molecules responsible for proinflammatory character of neutrophils, such as CD177 and OLMA4, are constitutively expressed on neutrophils leaving bone marrow. This idea is additionally supported by the finding of high reading density within PD-L1(CD274) gene promotor in H3K4me3-marked histone in neutrophils stimulated by IL-10 in comparison to LPS, TNF-α, or n.s. neutrophils. PD-L1(CD274) molecule, which is present on the surface of neutrophils, exerts T-cell-mediated immunosuppression *via* cell-cell contact (3). Altogether, these results suggest that neutrophils are prepared for a proinflammatory reaction immediately after leaving bone marrow, while IL-10 can act as the triggering factor switching them into immunosuppressive status.

Finally, we highlighted 12 DNA binding sites within H3K4me3-marked histone responsible for neutrophil polarization into immunosuppressive cells, and 19 involved in neutrophil activation. On the basis of current literature data, six-transmembrane epithelial antigen of the prostate 3 (Steap3), and dual-specificity tyrosine-phosphorylation-regulated kinase 2 (Dyrk2) caught our attention as the factors mediating immunosuppression. Steap3 is involved in regulating iron homeostasis and inhibiting TLR4-mediated inflammatory responses in macrophages (59, 60). In turn, Dyrk2 inhibits the release of TNF-α and IL-1β *via* phospho-Akt and negatively regulates virus-induced type I interferon by promoting TBK1 degradation *via* Ser527 phosphorylation (61). These factors involved in neutrophil suppression and designated from H3K4me3 ChiP-Seq analysis can be potentially considered as the targets in immunotherapy following validation by independent studies. Since the proinflammatory profile of neutrophils is already determined in the bone marrow, it seems reasonable to consider immunomodulation therapy during myelopoiesis before neutrophils enter peripheral blood and become ‘armed’.

## Author Contribution

PP designed, performed, and analysed experiments (HC neutrophil isolation, Chromatin Immunoprecipitation, cytokine/chemokine/growth factors profiling assays in both groups, RNA isolation, mRNA concentration analysis, Multiple Gene Profiling Array). MN performed HC neutrophil isolation, Chromatin Immunoprecipitation, mRNA concentration analysis (Multiple Gene Profiling Microarray), the manuscript writing & editing. NL prepared the manuscript (review & editing). MK enrolled periodontitis patients and healthy volunteers. ZJ enrolled sepsis patients. MM enrolled NMOSD patients. JD performed NGS and bioinformatics analysis. SM and MW performed ICC analysis. PL supervised and designed the study and experiments, interpreted the data, and wrote the manuscript (original draft). All authors revised the manuscript.

## Fundings

This work was supported by the grant from the National Science Centre 2015/17/B/NZ6/04251

## Conflict of Interest

Author JD was employed by company Genomed SA.

The remaining authors declare that the research was conducted in the absence of any commercial or financial relationships that could be construed as a potential conflict of interest.

## Publisher’s Note

All claims expressed in this article are solely those of the authors and do not necessarily represent those of their affiliated organizations, or those of the publisher, the editors and the reviewers. Any product that may be evaluated in this article, or claim that may be made by its manufacturer, is not guaranteed or endorsed by the publisher.

## Supplementary Material

Supplementary Figure 1
**(A)** Phenotypic analysis of purified neutrophils used for ChIP-Seq experiments. Flow cytometry analysis of CD14, CD15, and CD16 in the population of isolated neutrophils. Double-positive cells for CD16 and CD15 expression were identified as neutrophils, cells with CD14^high^CD15^+^ expression as monocytes, and cells with CD16^-^CD15^+^ as eosinophils. **(B)** ChIP-Seq library quality control analysis. ChIP-Seq library was generated from 1ng ChIP DNA with 18 cycles of PCR. The size of the library distribution is approximately 200-300 bp. **(C)** ChIP-Seq quality control report. Left diagram presents the distribution of the number of readings within peaks. Right panel presents the plot of correlation between peaksets (repeatable samples of high quality were expected to cluster together in the heatmap). Evaluation of the distribution, as well as heatmap, showed that variability and range of the signal in peaks within a sample are suitable for further analysis, except for one sample (#:1.TNF) which was excluded from further analysis. The analysis was performed by ChIPQC:1.21.0 software (author: Tom Carroll, Wei Liu, Ines de Santiago, Rory Stark).

Supplementary Figure 2 IL-10 and LPS induce NF-κB subunit RelA translocation to the cell nucleus that corresponds with increased H3K4me3 level, whereas TNF-α induces NF-κB subunit RelA translocation to the cell nucleus that corresponds with increased H3Ac level. **(A)** An example of ICC (left panel) and fluorescence intensity of H3K4me3 and NF-κB RelA (right panel). Orange arrows show double-positive signals. **(B)** ICC analysis of H3Ac and NF-κB RelA. **(C)** H3K4me3 and H3Ac florescent intensity comparison between non-stimulated and stimulated by IL-10, LPS, or TNF-α neutrophils. The bars represent average fluorescence intensity ± SD and ± S.E.M. calculated from four independent experiments. **(D)** Overlap coefficient analysis of NF-κB *vs.* H3K4me3 and NF-κB *vs.* H3Ac. Data are presented as mean ± SD and ± S.E.M. **(E)** The resolution of the confocal fluorescence microscope is 17-23 times lower than the histone size, thus, only allowing us to deduce the nucleus regions in which NF-κB is located.

Supplementary Figure 3 Neutrophils stimulated by IL-10 and LPS, but not TNF-α, affected H3K4me3-marked histone associated genes related to ‘mTOR signalling pathway’ and hypoxia-induced factors. **(A)** Binding site overlap in the GO term ‘mTOR signalling pathway’ and the comparison of peak density of mTOR genes. **(B)** The comparison of peak density within HIF1 and HIF2α, which alter leukocyte metabolism in responses to hypoxia.

Supplementary Figure 4 Neutrophils stimulated by IL-10 affected H3K4me3-marked histone-associated genes related to Histone H1, but not caspase initiator. **(A)** Gene Ontology analysis of apoptotic processes. The graph presents the average values ± SD calculated from four independent experiments. **(B)** Binding site overlap in the GO terms ‘apoptotic process’ and ‘neutrophils apoptotic process’. **(C)** The example of comparison of peak density in the genes responsible for histone-1-induced DNA fragmentation revealed high density of DNA within histone H1-0 and H1-4 in neutrophils stimulated by IL-10. **(D)** The representative example of comparison of peak density within representative genes involved in the regulation of apoptosis: initiators of caspases -9, -10; BCL-2 and XIAP apoptotic inhibitors. € The example of comparison of peak density within TSSs of PARP1 genes.

Supplementary Figure 5 Neutrophils stimulated by IL-10, LPS, or TNF-α affected H3K4me3-marked histone-associated genes connected with immunosuppressive PDL-1 (CD274) positive neutrophils, but not with their heterogeneity determined by the presence of CD177 or OLFA4 molecules. The one representative example from four independent experiments, a comparison analysis of gene peak density within CD274, CD177, and OLFA4 - factors considered as the main differentiating neutrophils in ‘sub-populations’.

Supplementary Figure 6 Neutrophils stimulated *in vitro* by IL-10, LPS, or TNF-α as a model of different status of these cells during immune response, correspond well to the clinical state of neutrophils in various diseases. HC neutrophils stimulated by LPS correspond to circulating neutrophils in sepsis; TNF-α corresponds to NMOSD and IL-10 with neutrophils isolated from periodontitis. High individual R^2^ correlation index revealed a strong correlation between the *in vitro* model and the patient’s neutrophil status in the cytokine/chemokine/growth factor released to extracellular space **(A)** as well as in the mRNA levels of NF-κB-related genes **(B)**. Each point represents the mean calculated from four independent experiments (*in vitro* model) *vs.* mean calculated from adequate clinical status: three samples of NMOSD, six sepsis, or 12 periodontitis. **(C)** Similar to neutrophils stimulated *in vitro*, co-localization of NF-κB within H3K4me3-marked histone is observed in periodontal disease, sepsis, or NMOSD. **(D)** H3Ac high level is only observed in the nucleus of NMOSD neutrophils.

Supplementary Figure 7 Similar to *in vitro* model of inflammation, binding site overlap graph points to a few common DNA binding sites within H3K4me3-marked histone which differ state of neutrophils during NMOSD, sepsis, and periodontitis. **(A)**. Pie chart of H3K4me3-marked histone binding site distribution in comparison to different diseases. Binding Site Overlaps graph was performed based on the average value of two independent clinical cases. **(B)** Gene Ontology analysis confirmed data obtained for neutrophils stimulated by LPS, TNF-α, or IL-10. The main neutrophil GO processes related to their antimicrobial function, correspond with data from the *in vitro* model. A similar relationship is observed in GO NF-κB pathways.

Supplementary Table 1
**(A)** The profile of cytokines/chemokines/growth factors released by non-, LPS, TNF-α, or IL-10-stimulated neutrophils. Data are presented as means ± SD calculated from the four independent experiments. **(B)** The profile of cytokines/chemokines/growth factors released by neutrophils isolated from whole blood healthy volunteers, sepsis, NMOSD, and periodontitis patients. Data are presented as means ± SD.

Supplementary Table 2 The list of all identified peaks within H3K4me3 in non-, LPS, TNF-α, or IL-10-stimulated neutrophils; neutrophils isolated from healthy volunteers, sepsis, NMOSD, and periodontitis patients. All peaks were divided into logical subsets specific for non-, LPS, TNF-α, or IL-10-stimulated (*in vitro* model) or HC, sepsis, NMOSD, and periodontitis patients.

Supplementary Table 3 The list of all Gene Ontology (GO) processes that characterize non-, LPS, TNF-α, and IL-10-stimulated neutrophils; neutrophils isolated from patients with sepsis, NMOSD, periodontitis, and HC. GO was performed by analyses of biological processes, molecular function, cellular components as well as pathway interactions. Data are arranged from the highest statistically significant value to the lowest one.

Supplementary Table 4
**(A, B)**. The list of target genes in the GO terms: ‘mTOR signaling pathway’, ‘neutrophil activation’, ‘cytokines,’ and ‘apoptotic process’ divided into logical subsets specific for non-, LPS, TNF-α, or IL-10-stimulated neutrophils.

Supplementary Table 5 The list of all tested mRNA for genes associated with activation of neutrophils *via* NF-κB. Data are presented as the relative fold differences to n.s. neutrophils. ‘H3K4me3 TSS regions’ column presents genes that are positioned by H3K4me3. Total count readings ≥10, within the first intron, were set as the detection limit.
