# Supplementary figures and images for: Changes Within H3K4me3-Marked Histone Reveal Molecular Background of Neutrophil Functional Plasticity
Source: Front Immunol. 2022 Jun 10;13:906311. doi: 10.3389/fimmu.2022.906311 (PMC9229595; doi:10.3389/fimmu.2022.906311)

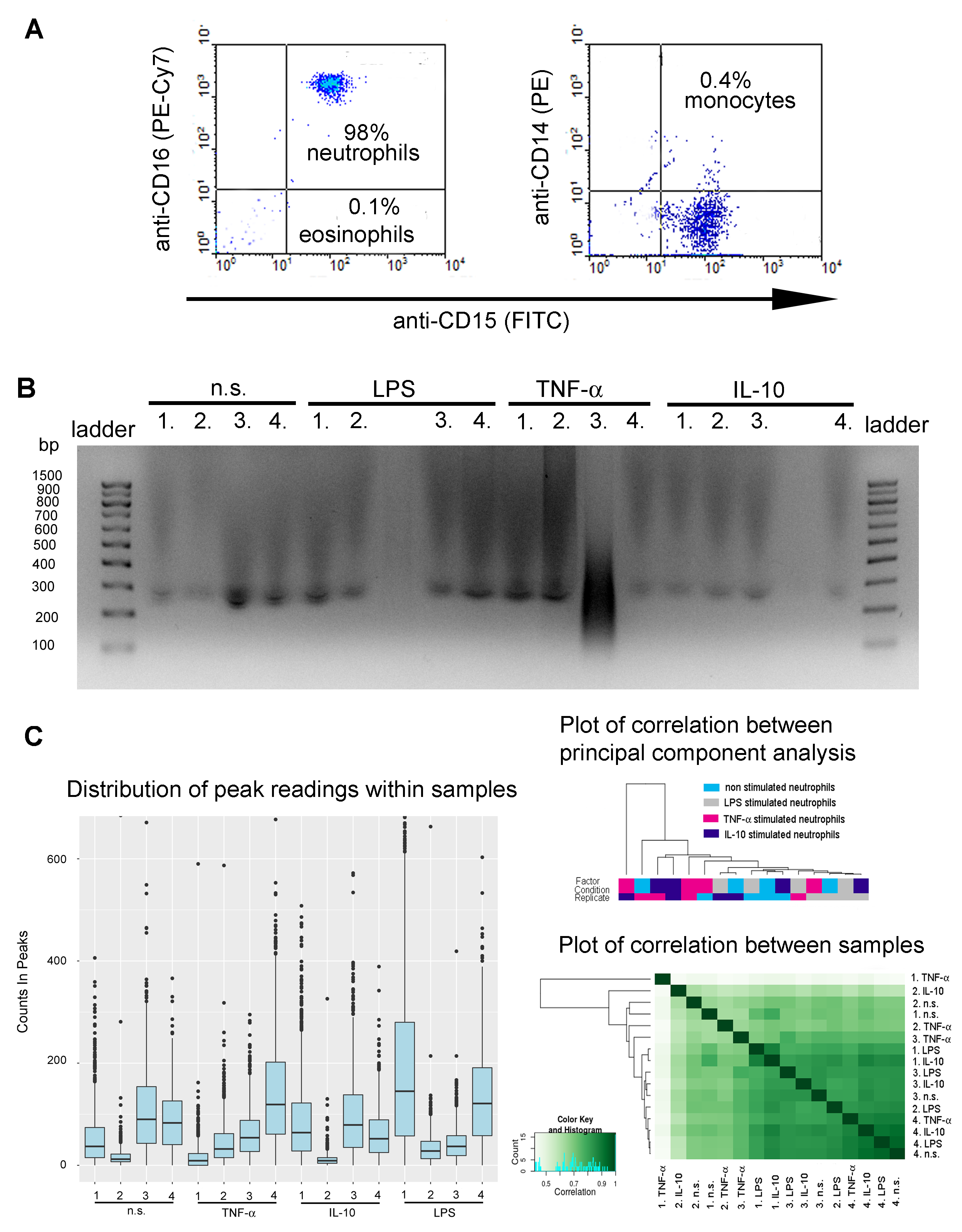

Supplement: Supplementary Figure 1 — (A) Phenotypic analysis of purified neutrophils used for ChIP-Seq experiments. Flow cytometry analysis of CD14, CD15, and CD16 in the population of isolated neutrophils. Double-positive cells for CD16 and CD15 expression were identified as neutrophils, cells with CD14highCD15+ expression as monocytes, and cells with CD16-CD15+ as eosinophils. (B) ChIP-Seq library quality control analysis. ChIP-Seq library was generated from 1ng ChIP DNA with 18 cycles of PCR. The size of the library distribution is approximately 200-300 bp. (C) ChIP-Seq quality control report. Left diagram presents the distribution of the number of readings within peaks. Right panel presents the plot of correlation between peaksets (repeatable samples of high quality were expected to cluster together in the heatmap). Evaluation of the distribution, as well as heatmap, showed that variability and range of the signal in peaks within a sample are suitable for further analysis, except for one sample (#:1.TNF) which was excluded from further analysis. The analysis was performed by ChIPQC:1.21.0 software (author: Tom Carroll, Wei Liu, Ines de Santiago, Rory Stark). [file Image_1.tif]

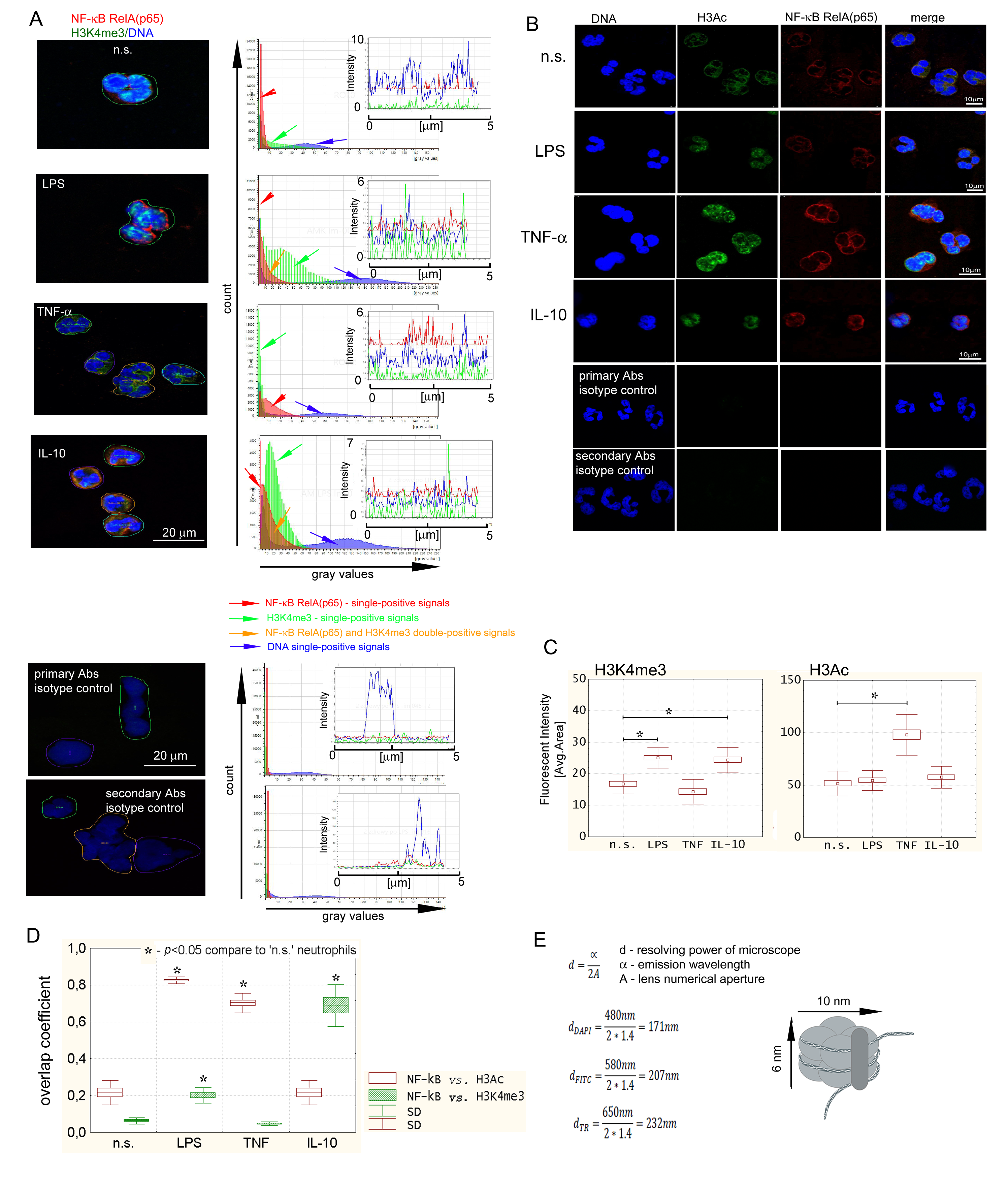

Supplement: Supplementary Figure 2 — IL-10 and LPS induce NF-κB subunit RelA translocation to the cell nucleus that corresponds with increased H3K4me3 level, whereas TNF-α induces NF-κB subunit RelA translocation to the cell nucleus that corresponds with increased H3Ac level. (A) An example of ICC (left panel) and fluorescence intensity of H3K4me3 and NF-κB RelA (right panel). Orange arrows show double-positive signals. (B) ICC analysis of H3Ac and NF-κB RelA. (C) H3K4me3 and H3Ac florescent intensity comparison between non-stimulated and stimulated by IL-10, LPS, or TNF-α neutrophils. The bars represent average fluorescence intensity ± SD and ± S.E.M. calculated from four independent experiments. (D) Overlap coefficient analysis of NF-κB vs. H3K4me3 and NF-κB vs. H3Ac. Data are presented as mean ± SD and ± S.E.M. (E) The resolution of the confocal fluorescence microscope is 17-23 times lower than the histone size, thus, only allowing us to deduce the nucleus regions in which NF-κB is located. [file Image_2.tif]

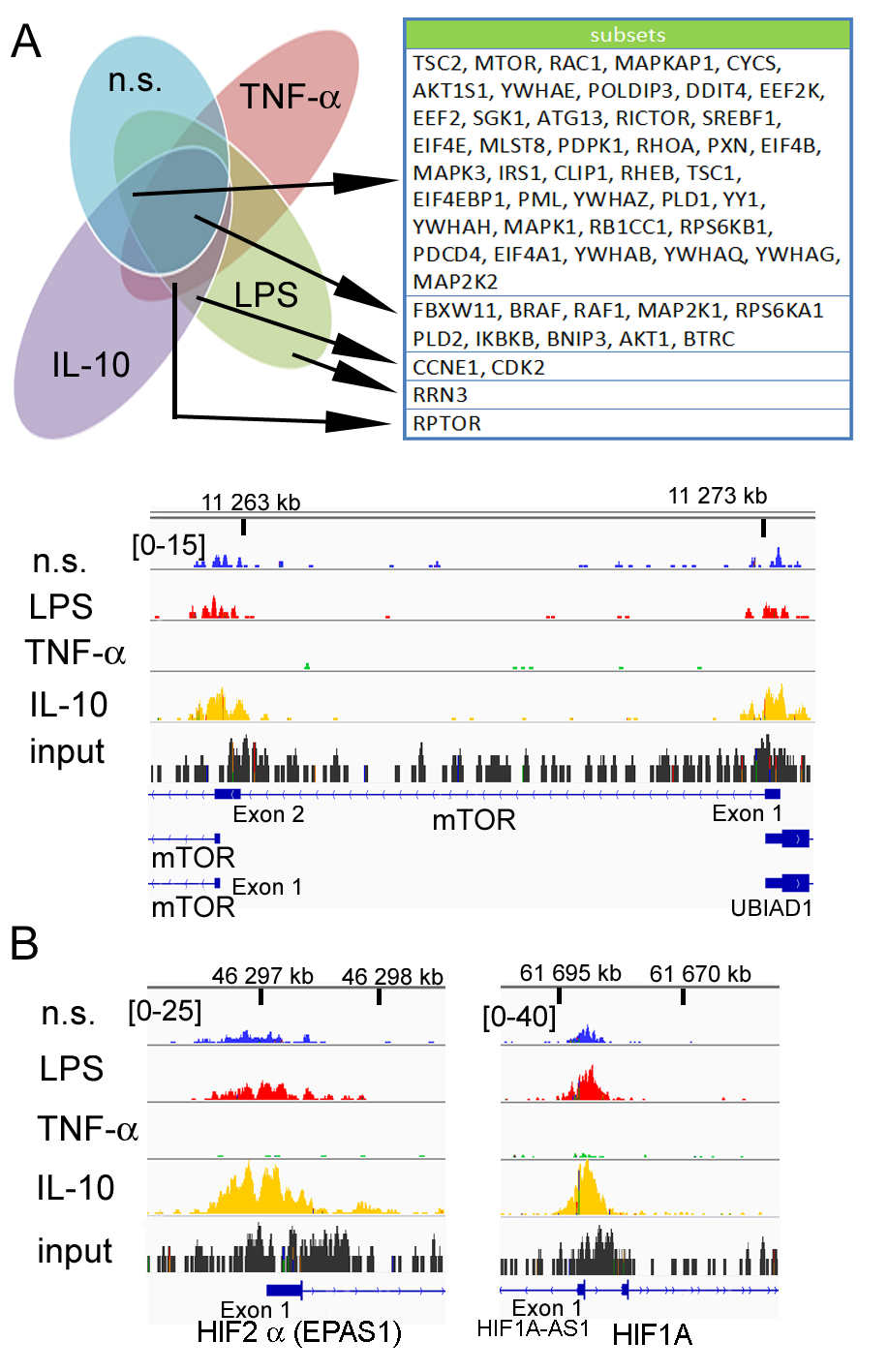

Supplement: Supplementary Figure 3 — Neutrophils stimulated by IL-10 and LPS, but not TNF-α, affected H3K4me3-marked histone associated genes related to ‘mTOR signalling pathway’ and hypoxia-induced factors. (A) Binding site overlap in the GO term ‘mTOR signalling pathway’ and the comparison of peak density of mTOR genes. (B) The comparison of peak density within HIF1 and HIF2α, which alter leukocyte metabolism in responses to hypoxia. [file Image_3.tif]

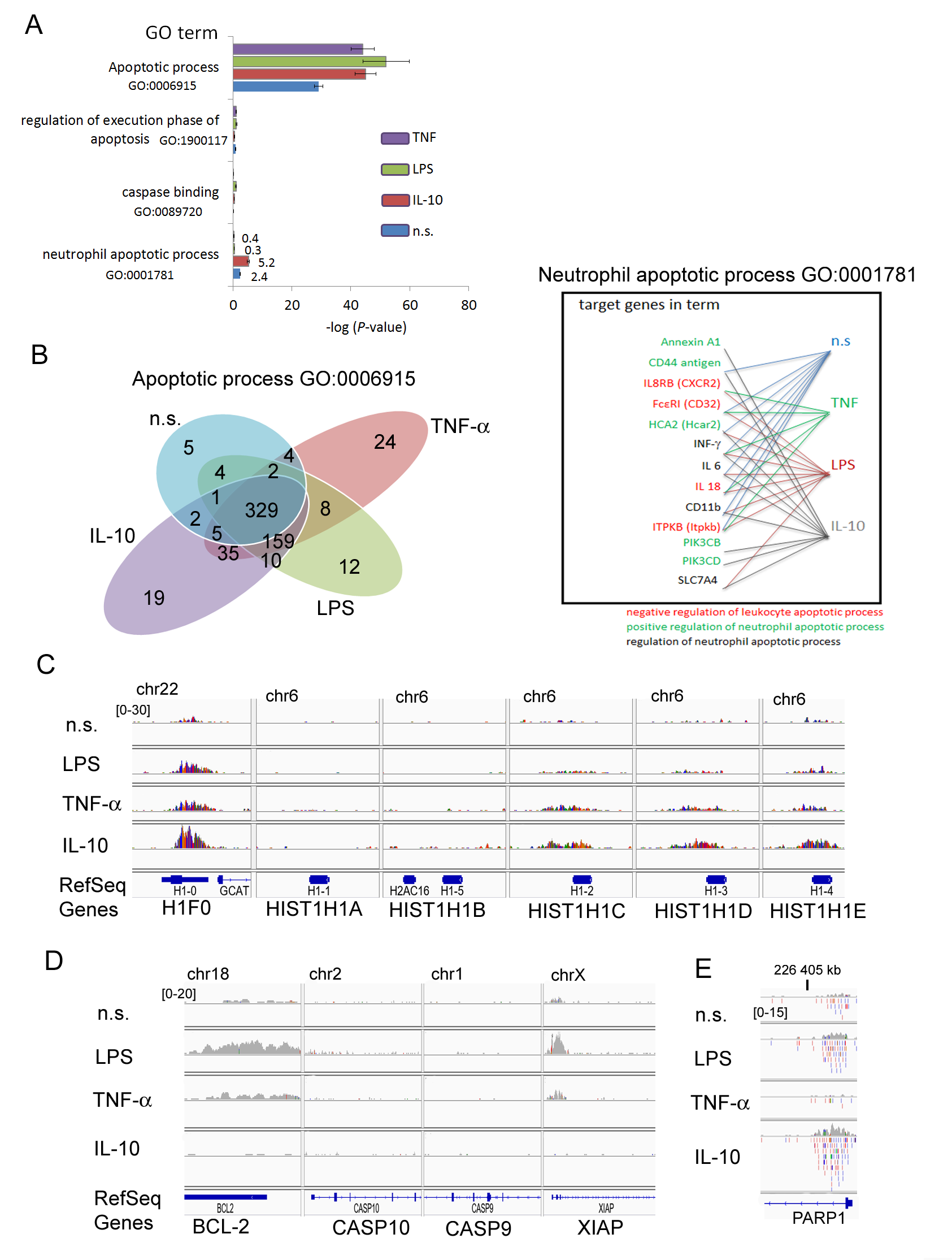

Supplement: Supplementary Figure 4 — Neutrophils stimulated by IL-10 affected H3K4me3-marked histone-associated genes related to Histone H1, but not caspase initiator. (A) Gene Ontology analysis of apoptotic processes. The graph presents the average values ± SD calculated from four independent experiments. (B) Binding site overlap in the GO terms ‘apoptotic process’ and ‘neutrophils apoptotic process’. (C) The example of comparison of peak density in the genes responsible for histone-1-induced DNA fragmentation revealed high density of DNA within histone H1-0 and H1-4 in neutrophils stimulated by IL-10. (D) The representative example of comparison of peak density within representative genes involved in the regulation of apoptosis: initiators of caspases -9, -10; BCL-2 and XIAP apoptotic inhibitors. € The example of comparison of peak density within TSSs of PARP1 genes. [file Image_4.tif]

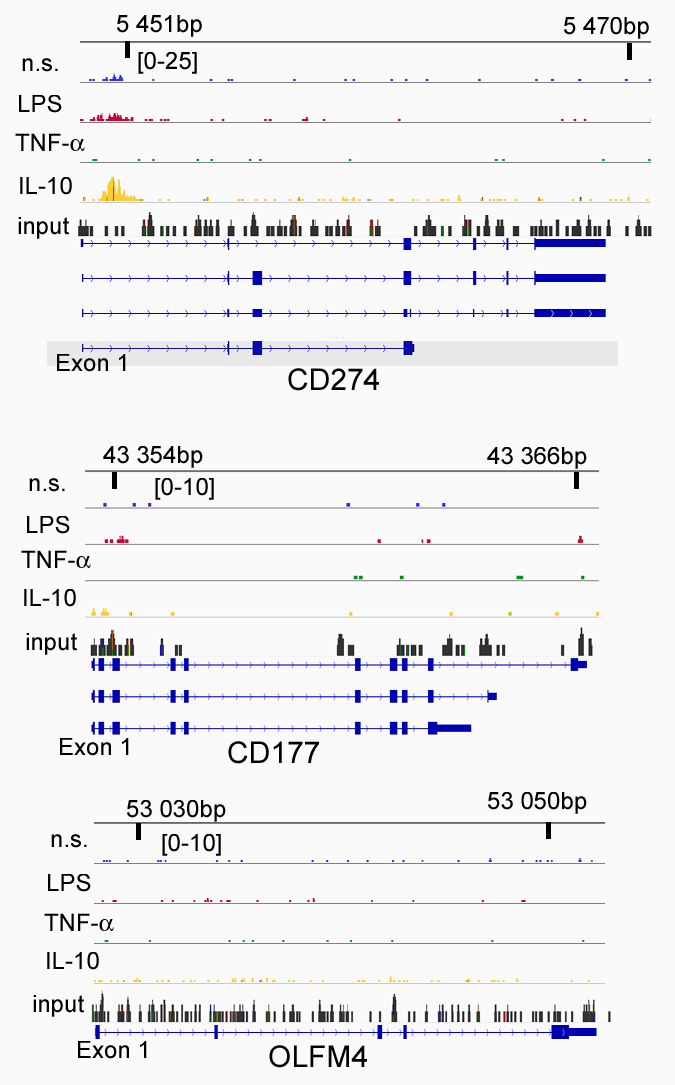

Supplement: Supplementary Figure 5 — Neutrophils stimulated by IL-10, LPS, or TNF-α affected H3K4me3-marked histone-associated genes connected with immunosuppressive PDL-1 (CD274) positive neutrophils, but not with their heterogeneity determined by the presence of CD177 or OLFA4 molecules. The one representative example from four independent experiments, a comparison analysis of gene peak density within CD274, CD177, and OLFA4 - factors considered as the main differentiating neutrophils in ‘sub-populations’. [file Image_5.tif]

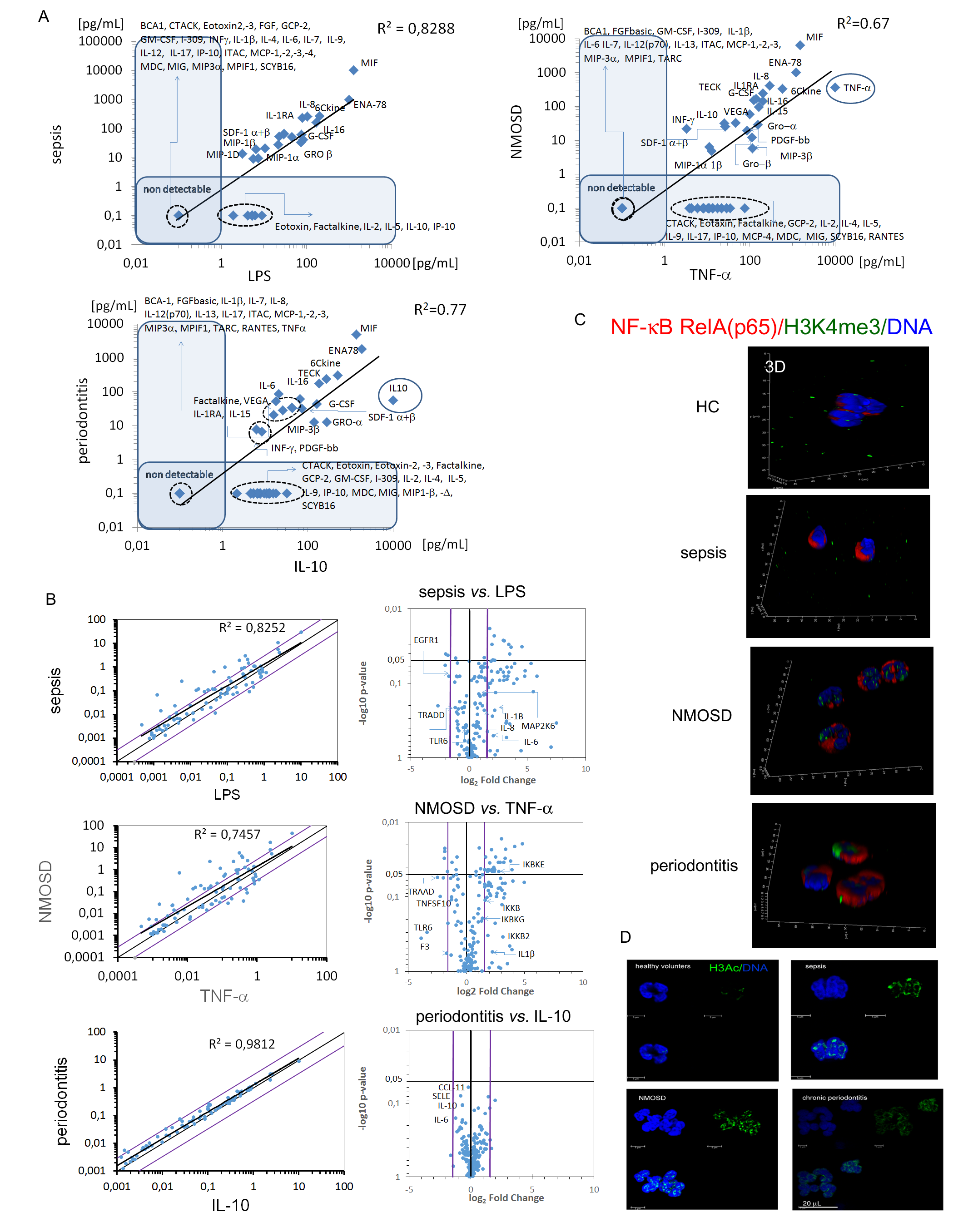

Supplement: Supplementary Figure 6 — Neutrophils stimulated in vitro by IL-10, LPS, or TNF-α as a model of different status of these cells during immune response, correspond well to the clinical state of neutrophils in various diseases. HC neutrophils stimulated by LPS correspond to circulating neutrophils in sepsis; TNF-α corresponds to NMOSD and IL-10 with neutrophils isolated from periodontitis. High individual R2 correlation index revealed a strong correlation between the in vitro model and the patient’s neutrophil status in the cytokine/chemokine/growth factor released to extracellular space (A) as well as in the mRNA levels of NF-κB-related genes (B). Each point represents the mean calculated from four independent experiments (in vitro model) vs. mean calculated from adequate clinical status: three samples of NMOSD, six sepsis, or 12 periodontitis. (C) Similar to neutrophils stimulated in vitro, co-localization of NF-κB within H3K4me3-marked histone is observed in periodontal disease, sepsis, or NMOSD. (D) H3Ac high level is only observed in the nucleus of NMOSD neutrophils. [file Image_6.tif]

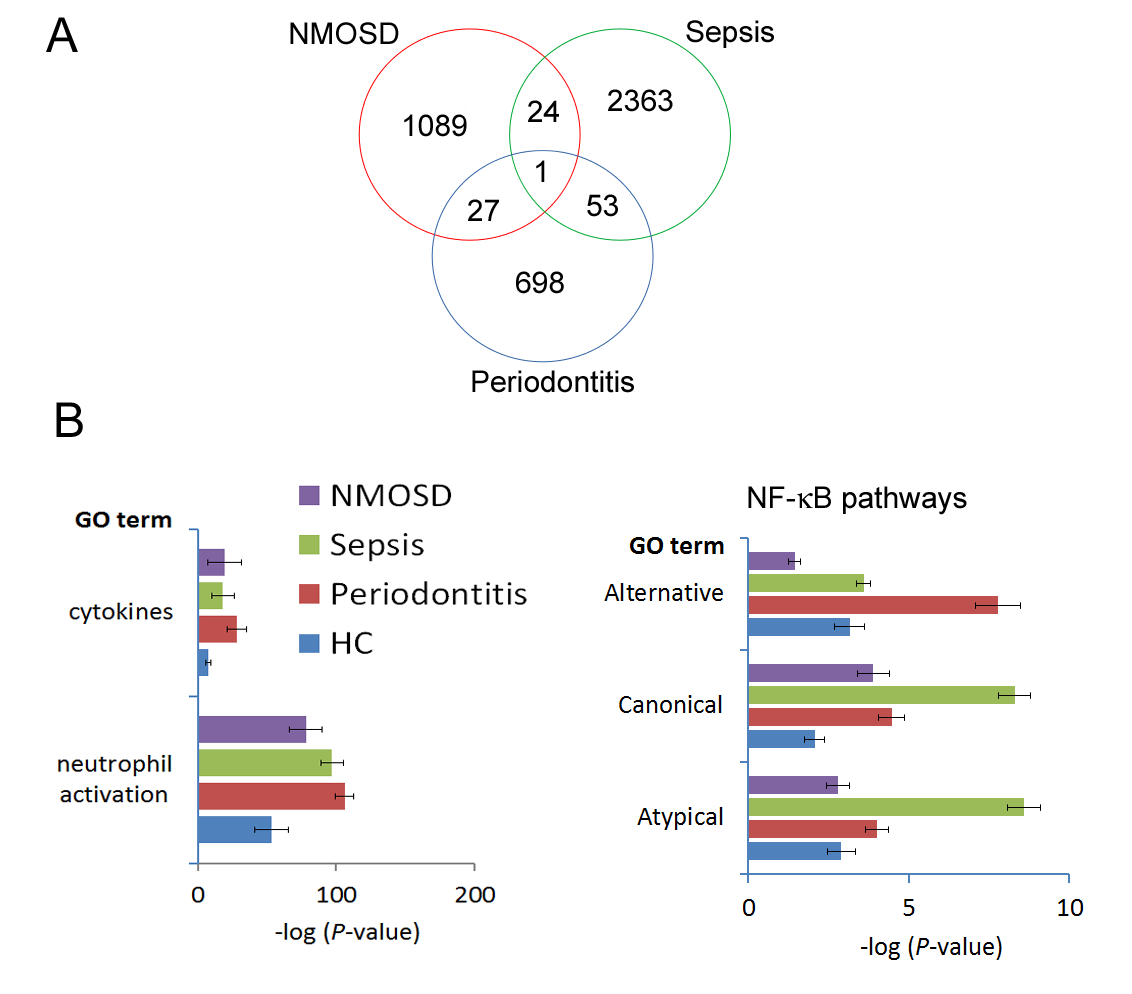

Supplement: Supplementary Figure 7 — Similar to in vitro model of inflammation, binding site overlap graph points to a few common DNA binding sites within H3K4me3-marked histone which differ state of neutrophils during NMOSD, sepsis, and periodontitis. (A). Pie chart of H3K4me3-marked histone binding site distribution in comparison to different diseases. Binding Site Overlaps graph was performed based on the average value of two independent clinical cases. (B) Gene Ontology analysis confirmed data obtained for neutrophils stimulated by LPS, TNF-α, or IL-10. The main neutrophil GO processes related to their antimicrobial function, correspond with data from the in vitro model. A similar relationship is observed in GO NF-κB pathways. [file Image_7.tif]

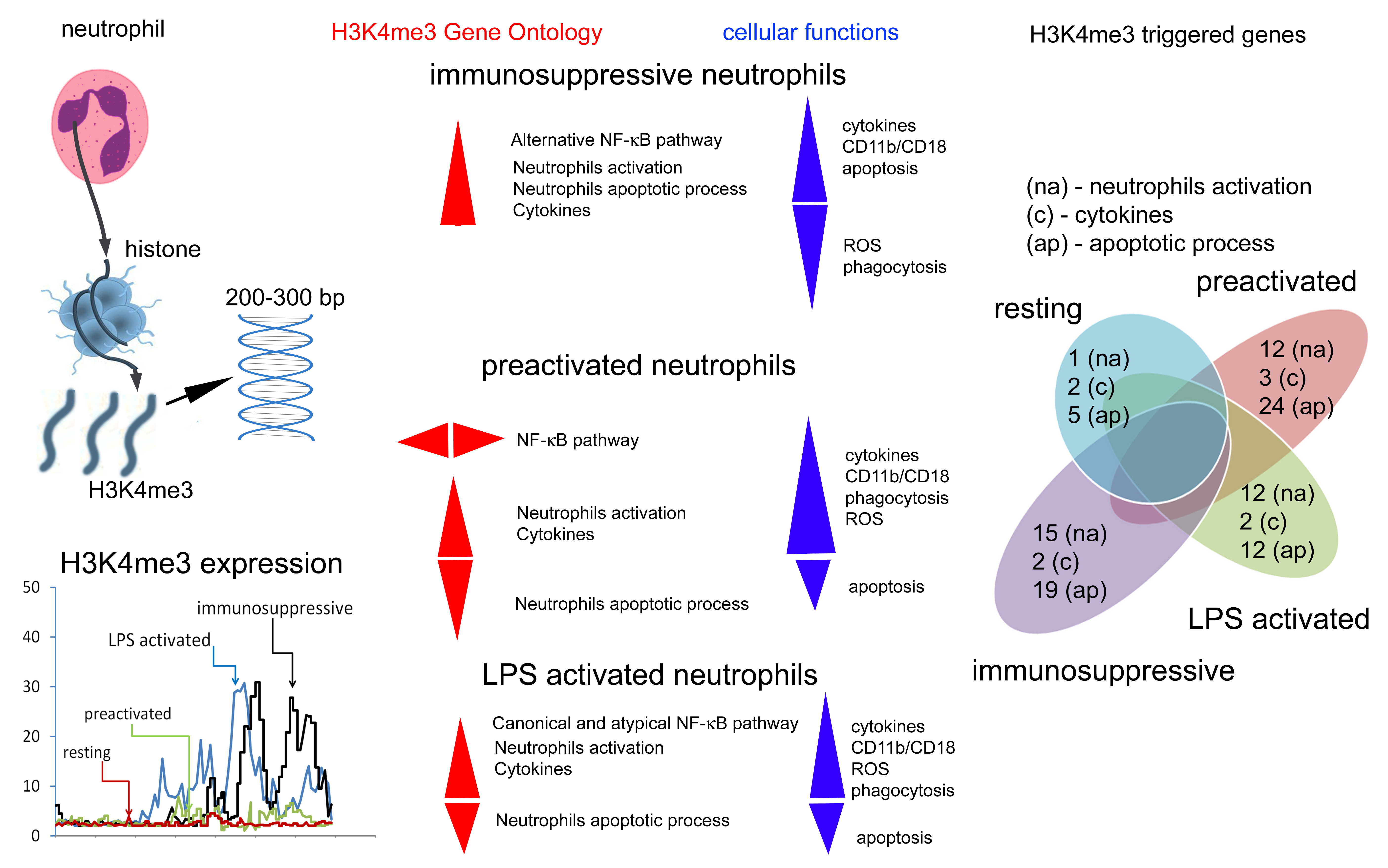

Supplement: Supplementary file 13 [file Image_8.tif]
