# Supplementary material for: Changes Within H3K4me3-Marked Histone Reveal Molecular Background of Neutrophil Functional Plasticity
Source: Front Immunol. 2022 Jun 10;13:906311. doi: 10.3389/fimmu.2022.906311 (PMC9229595; doi:10.3389/fimmu.2022.906311)
Supplement: Supplementary Table 1 — (A) The profile of cytokines/chemokines/growth factors released by non-, LPS, TNF-α, or IL-10-stimulated neutrophils. Data are presented as means ± SD calculated from the four independent experiments. (B) The profile of cytokines/chemokines/growth factors released by neutrophils isolated from whole blood healthy volunteers, sepsis, NMOSD, and periodontitis patients. Data are presented as means ± SD. [file Table_1.docx]

Supplementary Table S1A. The profile of cytokines/chemokines/growth factors released by non-, LPS, TNF-α or IL-10-stimulated neutrophils. Data are presented in pg/mL as means ± SD calculated based on the four independent experiments.

|  | **ns** | **LPS** | **TNF-α** | **IL-10** |
| --- | --- | --- | --- | --- |
| **6Ckine (CCL21)** | 101.5 ± 41.03 | 202.4 ± 46.74 ^a^ | 577.8 ± 65.98 ^a^ | 506.6 ± 59.48 ^a,^ ^b^ |
| **BCA-1 (CXCL13)** | nd | nd | nd | nd |
| **CTACK (CCL27)** | nd | nd | 5.9 ± 0.94 ^a^ | 7.73 ± 0.36 ^a, b^ |
| **ENA-78 (CXCL5)** | 671.5 ± 458.44 | 999.0 ± 271.22 ^a^ | 1197.9 ± 212.37 ^a^ | 1848.1 ± 129.62 ^a, b, c^ |
| **Eotaxin (CCL11)** | nd | 6.2 ± 1.12 | 10.3 ± 1.12 ^a^ | 12.6 ± 1.84 ^a^ |
| **Eotaxin-2 (CCL24)** | nd | nd | 7.96 ± 1.52 ^a^ | 7.22 ± 0.35 ^a, b^ |
| **Eotaxin-3 (CCL26)** | nd | nd | 13.6 ± 3.06 ^a^ | 11.8 ± 1.03 ^a, b^ |
| **Factalkine (CX3CL1)** | nd | 5.3 ± 2.78 | 10.6 ± 4.43 ^a^ | 42.5 ± 3.36 ^a, b, c^ |
| **FGFbasic** | nd | nd | nd | nd |
| **GCP-2 (CXCL6)** | nd | nd | 8.1 ± 2.17 ^a^ | 5.4 ± 4.24 |
| **G-CSF** | nd | 83.5 ± 57.64 ^a^ | 122.7 ± 49.78 ^a^ | 162.0 ± 40.80 ^a^ |
| **GM-CSF** | nd | nd | 3.83 ± 0.68 | 6.5 ± 0.52 ^a^ |
| **Gro-α (CXCL1)** | nd | 73.5 ± 39.17 ^a^ | 155.7 ± 53.74 ^a^ | 141.6 ± 16.24 ^a^ |
| **Gro-β (CXCL2)** | 26.6 ± 18.6 | 76.2 ± 45.85 ^a^ | 111.8 ± 58.16 ^a^ | 43.5 ± 17.99 ^a, b, c^ |
| **I-309 (CCL-1)** | nd | nd | 21.6 ± 3.22 ^a^ | nd ^c^ |
| **IFN-γ** | nd | nd | 3.3 ± 0.49 | nd ^c^ |
| **IL-1β** | nd | nd | nd | nd |
| **IL-1RA** | 66.7 ± 43.70 | 80.5 ± 28.34 | 139.8 ± 40.53 ^a^ | 35.9 ± 49.66 ^b, c^ |
| **IL-2** | n.d. | 4.2 ± 1.34 | 34.6 ± 2.64 ^a^ | 9.5 ± 1.01 ^c^ |
| **IL-4** | nd | nd | 8.3 ± 1.49 | 10.7 ± 2.63 ^a^ |
| **IL-5** | 3.5 ± 0.84 | 1.9 ± 0.89 | 28.7 ± 5.99 ^a^ | 4.7 ± 1.67 ^c^ |
| **IL-6** | nd | nd | nd | 20.6 ± 1.73 ^a, b, c^ |
| **IL-7** | nd | nd | nd | nd |
| **IL-8 (CXCL8)** | nd | 103.4 ± 36.83 ^a^ | 290.1 ± 118.11 ^a^ | 23.9 ± 3.79 ^a, b, c^ |
| **IL-9** | nd | nd | 13.4 ± 2.96 ^a^ | n.d. ^c^ |
| **IL-10** | nd | nd | nd | >10000 ^a, b, c^ |
| **IL-12(p70)** | nd | nd | nd | nd |
| **IL-13** | nd | nd | nd | nd |
| **IL-15** | 23.5 ± 7.64 | 22.7 ± 6.91 | 162.4 ± 14.99 ^a^ | 26.1 ± 10.38 ^c^ |
| **IL-16** | 157.1 ± 97.79 | 170.5 ± 82.56 | 201.1 ± 116.90 | 184.5 ± 105.80 |
| **IL-17** | nd | nd | 21.9 ± 5.81 ^a^ | nd ^c^ |
| **IP-10 (CXCL10)** | nd | 1.2 ± 4.90 | 17.3 ± 2.92 ^a^ | 15.6 ± 4.66 ^a^ |
| **I-TAC (CXCL11)** | nd | nd | nd | nd |
| **MCP-1 (CCL2)** | nd | nd | nd | nd |
| **MCP-2 (CCL8)** | nd | nd | nd | nd |
| **MCP-3 (CCL7)** | nd | nd | nd | nd |
| **MCP-4 (CCL13)** | nd | nd | 4.3 ± 1.23 ^a^ | 5.3 ± 1.74 ^a, b^ |
| **MDC (CCL22)** | nd | nd | 76.5 ± 43.19 ^a, b^ | 32.3 ± 47.27 ^c^ |
| **MIF (*Macrophage migration inhibitory factor*)** | 863.6 ± 617.79 | 1268.7 ± 545.99 ^a^ | 1500.2 ± 540.45 ^a^ | 1382.8 ± 497.71 ^a^ |
| **MIG (CXCL9)** | nd | nd | 11.1 ± 4.98 ^a, b^ | 6.44 ± 5.19 |
| **MIP-1α (CCL3)** | nd | 10.7 ± 4.58 ^a^ | 12.7 ± 10.44 | 15.9 ± 29.19 |
| **MIP 1β (CCL4)** | nd | 6.5 ± 1.81 ^a^ | 26.6 ± 15.70 ^a^ | 2.2 ± 0.99 ^a, c^ |
| **MIP-1Δ (CCL15)** | nd | 3.1 ± 2.05 | 11.1 ± 2.29 ^a^ | 12.8 ± 6.19 ^a^ |
| **MIP-3α (CCL20)** | nd | nd | nd | nd |
| **MIP-3β (CCL19)** | nd | 7.4 ± 2.92 | 114.1 ± 166.07 ^b^ | 280.5 ± 161.26 ^a, b, c^ |
| **MPIF-1 (CCL23)** | nd | nd | nd | nd |
| **PDGF-bb** | 4.9 ± 3.92 | 5.7 ± 4.48 | 86.0 ± 2.54 ^a, b^ | 6.3 ± 4.73 ^c^ |
| **SCYB16 (CXCL16)** | nd | nd | 4.23 ± 1.82 ^a, b^ | 4.6 ± 1.89 ^a, b^ |
| **SDF-1α+β (CXCL12)** | 15.6 ± 3.92 | 21.9 ± 0.52 ^a^ | 46.7 ± 24.23 ^a^ | 72.7 ± 24.10 ^a, b, c^ |
| **TARC (CCL17)** | nd | nd | nd | nd |
| **TECK (CCL25)** | 32.0 ± 8.63 | 44.8 ± 8.63 | 203.6 ± 53.96 ^a, b^ | 271.55 ± 41.72 ^a, b, c^ |
| **RANTES (CCL5)** | nd | nd | 28.2 ± 1.23 ^a, b^ | nd ^c^ |
| **TNF-α** | nd | 29.9 ± 10.10 ^a^ | >10000 ^a, b^ | nd ^c, b^ |
| **VEGF** | nd | 28.9 ± 12.91 ^a^ | 99.0 ± 15.03 ^a, b^ | 33.2 ± 10.62 ^a, c^ |

a - statistically significant differences to non-stimulated neutrophils; b - statistically significant differences to LPS-stimulated neutrophils; c - statistically significant differences to TNF-α-stimulated neutrophils. nd – non detectable

Supplementary Table S1B**.** The profile of cytokines/chemokines/growth factors released by neutrophils isolated from sepsis, NMOSD, periodontitis patients and healthy volunteers (HC). Data are presented in pg/mL as means ± SD.

|  | **HC** | **sepsis** | **NMOSD** | **periodontitis** |
| --- | --- | --- | --- | --- |
|  | **n=4** | **n=6** | **n=3** | **n=12** |
| **6Ckine (CCL21)** | 93.05 ± 19.96 | 274.0 ± 23.91* | 329.9 ± 83.09* | 307.5 ± 72.9* |
| **BCA-1 (CXCL13)** | nd | nd | nd | nd |
| **CTACK (CCL27)** | nd | nd | nd | nd |
| **ENA-78 (CXCL5)** | 841.9 ± 149.70 | 991.9 ± 172.87 | 1030.1 ± 200.07 | 1839.3 ± 341.09* |
| **Eotaxin (CCL11)** | nd | nd | nd | nd |
| **Eotaxin-2 (CCL24)** | nd | nd | nd | nd |
| **Eotaxin-3 (CCL26)** | nd | nd | nd | nd |
| **Factalkine (CX3CL1)** | nd | nd | nd | 34.6 ± 14.43* |
| **FGFbasic** | nd | nd | nd | nd |
| **GCP-2 (CXCL6)** | nd | nd | nd | nd |
| **G-CSF** | nd | 42.3 ± 45.90 | 153.6 ± 34.01* | 43.5 ± 14.98* |
| **GM-CSF** | nd | nd | nd | nd |
| **Gro-α (CXCL1)** | nd | 33.8 ± 33.62 | 29.4 ± 0.00 | 12.6 ± 4.57 |
| **Gro-β (CXCL2)** | 23.5 ± 8.21 | 62.0 ± 14.51 | 12.4 ± 9.80* | 33.8 ± 20.31 |
| **I-309 (CCL-1)** | nd | nd | nd | nd |
| **IFN-γ** | nd | nd | 22.1 ± 0.68* | 6.6 ± 2.64* |
| **IL-1β** | nd | nd | nd | nd |
| **IL-1RA** | 54.1 ± 29.96 | 235.1 ± 215.63* | 171.1 ± 84.06* | 61.7 ± 38.17 |
| **IL-2** | nd | nd | nd | nd |
| **IL-4** | nd | nd | nd | nd |
| **IL-5** | 2.9 ± 0.99 | nd | nd | nd |
| **IL-6** | nd | nd | nd | 85.4 ± 21.76 |
| **IL-7** | nd | nd | nd | nd |
| **IL-8 (CXCL8)** | nd | 260.9 ± 45.12* | 414.8 ± 54.49* | nd |
| **IL-9** | nd | nd | nd | nd |
| **IL-10** | nd | nd | 32.1 ± 12.93 | 55.7 ± 12.59* |
| **IL-12(p70)** | nd | nd | nd | nd |
| **IL-13** | nd | nd | nd | nd |
| **IL-15** | 24.1 ± 6.13 | 52.8 ± 7.79* | 98.1 ± 29.53* | 28.7 ± 19.49 |
| **IL-16** | 107.8 ± 67.89 | 165.8 ± 29.45* | 245.2 ± 87.50* | 176.6 ± 121.90* |
| **IL-17** | nd | nd | nd | nd |
| **IP-10 (CXCL10)** | nd | nd | nd | nd |
| **I-TAC (CXCL11)** | nd | nd | nd | nd |
| **MCP-1 (CCL2)** | nd | nd | nd | nd |
| **MCP-2 (CCL8)** | nd | nd | nd | nd |
| **MCP-3 (CCL7)** | nd | nd | nd | nd |
| **MCP-4 (CCL13)** | nd | nd | nd | nd |
| **MDC (CCL22)** | nd | nd | nd | nd |
| **MIF (*Macrophage migration inhibitory factor*)** | 1033.4 ± 415.19 | 10135.2 ± 2699.11* | 6469.7 ± 3486.62* | 4943.1 ± 3814.02* |
| **MIG (CXCL9)** | nd | nd | nd | nd |
| **MIP-1α (CCL3)** | nd | 20.7 ± 20.99 | 4.8 ± 2.27 | 20.8 ± 10.80 |
| **MIP 1β (CCL4)** | nd | 19.3 ± 9.74 | 26.1 ± 10.50 | n.d. |
| **MIP-1Δ (CCL15)** | 7.2 ± 4.19 | 13.5 ± 9.05 | 6.5 ± 0.86 | nd |
| **MIP-3α (CCL20)** | nd | nd | nd | nd |
| **MIP-3β (CCL19)** | 11.1 ± 9.62 | 9.5 ± 8.33 | 5.8 ± 1.87 | 2.6 ± 2.80 |
| **MPIF-1 (CCL23)** | nd | nd | nd | nd |
| **PDGF-bb** | 6.9 ± 4.57 | 9.2 ± 3.00 | 19.7 ± 5.02* | 7.7 ± 2.59 |
| **SCYB16 (CXCL16)** | nd | nd | nd | nd |
| **SDF-1α+β (CXCL12)** | 38.4 ± 13.55 | 28.3 ± 10.42 | 32.8 ± 12.11 | 32.3 ± 12.98 |
| **TARC (CCL17)** | nd | nd | nd | nd |
| **TECK (CCL25)** | 45.5 ± 7.87 | 51.7 ± 16.62 | 143.9 ± 16.47* | 238.7 ± 88.05* |
| **RANTES (CCL5)** | nd | nd | nd | nd |
| **TNF-α** | nd | 66.0 ± 23.37* | 366.0 ± 103.37* | nd |
| **VEGF** | nd | 63.6 ± 15.31* | 60.1 ± 13.63* | 74.7 ± 30.15* |

* - statistically significant differences compare to HC; . nd – non detectable
