# Supplementary material for: Changes Within H3K4me3-Marked Histone Reveal Molecular Background of Neutrophil Functional Plasticity
Source: Front Immunol. 2022 Jun 10;13:906311. doi: 10.3389/fimmu.2022.906311 (PMC9229595; doi:10.3389/fimmu.2022.906311)
Supplement: Supplementary Table 4 — (A, B). The list of target genes in the GO terms: ‘mTOR signaling pathway’, ‘neutrophil activation’, ‘cytokines,’ and ‘apoptotic process’ divided into logical subsets specific for non-, LPS, TNF-α, or IL-10-stimulated neutrophils. [file Table_4.docx]

Supplementary Tabel S4 A.


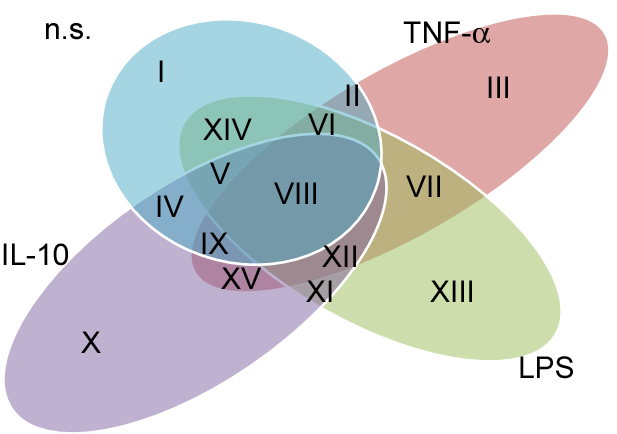


|  | GO terms |
| --- | --- |
| subsets | **mTOR signaling pathway** |
| I | ud |
| II | ud |
| III | ud |
| IV | ud |
| V | TSC2, MTOR, RAC1, MAPKAP1, CYCS, AKT1S1, YWHAE, POLDIP3, DDIT4, EEF2K, EEF2, SGK1, ATG13, RICTOR, SREBF1, EIF4E, MLST8, PDPK1, RHOA, PXN, EIF4B, MAPK3, IRS1, CLIP1, RHEB, TSC1, EIF4EBP1, PML, YWHAZ, PLD1, YY1, YWHAH, MAPK1, RB1CC1, RPS6KB1, PDCD4, EIF4A1, YWHAB, YWHAQ, YWHAG, MAP2K2 |
| VI | ud |
| VII | ud |
| VIII | FBXW11, BRAF, RAF1, MAP2K1, RPS6KA1 |
| IX | ud |
| X | ud |
| XI | PLD2, IKBKB, BNIP3, AKT1, BTRC |
| XII | CCNE1, CDK2 |
| XIII | RRN3 |
| XIV | ud |
| XV | RPTOR |

ud – undetectable

Supplementary Table S4 B

|  | GO terms | | |
| --- | --- | --- | --- |
| subsets | **Neutrophils activation**  **GO:0042119** | **Cytokines**  **GO:0000910** | **Apoptotic process**  **GO:0006915** |
| I | B4GALT1 | CHMP1A, NUSAP1 | EPB41L3, DAB2IP, IL6R, BECN1, C3orf38 |
| II | ud | ud | MYC, BAG6, ATF2, DDIAS |
| III | CD177, NHLRC3, BST2, LAIR1, JUP, DSP, ANXA2, KMT2E, PSMC2, NDUFC2, IL15, PPIE | CHMP5, SEPTIN11, PLK1, MITD1 | PDE1B, DCC, TOPORS, PRDX5, LALBA, CEACAM5, SNW1, THEM4, DDX47, NCKAP1, XKR7, SEMA6A, POU4F2, DLC1, CD5, CSNK2A2, SNCA, PHLDA2, YARS, CDCA7, PTRH2, PAWR, DUX4, UACA |
| IV | ud | ud | TNFAIP3, DIABLO |
| V | KCNAB2, TBC1D10C, PGRMC1 | ARL3, TRIM36 | P2RX1, EXOC5, UBE2Z, ZDHHC16, KANK2 |
| VI | DNASE1L1 | SON | CIAPIN1, IRF3 |
| VII | SYNGR1 | ALKBH4 | TRIM39, NTN1, BIRC3, TNFAIP1, EXOG, MOAP1, THOC6, NLRP3 |
| VIII | LAMP1, GAA, ADAM10, ATP8A1, DNAJC3, HEXB, S100A8, GYG1, HMOX2, CMTM6, PA2G4, PSMD1, QPCT, AP1M1, ACTR2, LAMP2, NFAM1, ARHGAP45, HSPA8, CDA, C5AR1, CSTB, PTGES2, DDX3X, ALDOA, CFD, STXBP3, SERPINB6, ATP6V0C, ADAM8, F2RL1, HMGB1, DNAJC5, GMFG, APEH, PSEN1, TMEM179B, GNS, CD59, ARPC5, GDI2, BIN2, OSTF1, SDCBP, RNASET2, DGAT1, CTSD, LPCAT1, PSMD12, FTL, QSOX1, PNP, DYNLT1, ANO6, PSMC3, RAP1B, PYGL, VAMP2, NPC2, PKM, HGSNAT, CCT8, RAB27A, DYNC1H1, CAT, TLR2, YPEL5, CR1, ACLY, FCGR2A, ADGRE5, SERPINB1, LRG1, RAB5C, SLC15A4, RHOG, SCAMP1, S100A11, TUBB4B, IQGAP1, RAC1, NIT2, FTH1, RHOF, ARSA, ALOX5,, LAMTOR2, S100P, BRI3, TMBIM1, CPNE1, CCT2, PGLYRP1, PADI2, CXCR2, PREX1, ATP6V0A1, HLA-H, STK10, DOK3, LAMTOR1, DSN1, DDOST, RAB24, XRCC6, CXCL1, PDXK, MANBA, RAP2B, B2M, CTSZ, EEF1A1, PPIA, CD58, CTSC, HUWE1, SLC44A2, CNN2, S100A9, RAB3D, HEBP2, ATP11A, PYGB, TNFRSF1B, PTPRC, FGR, DEGS1, PSAP, PTAFR, FAF2, ARL8A, MAPK14, KCMF1, AMPD3, AP2A2, RAP1A, NFKB1, ATP6AP2, CD55, ACTR10, CXCL6, CYSTM1, COTL1, RAB5B, VCP, HSPA6, ITGB2, GSN, CYBA, HPSE, AGPAT2, IST1, CAB39, PAFAH1B2, ROCK1, RAB37, LILRB3, CPPED1, GRN, ACTR1B, PSMD7, ASAH1, CDK13, RAB6A, HVCN1, EEF2, PYCARD, SLCO4C1, CKAP4, PSMA5, FOLR3, IGF2R, SLC2A3, CREG1, ARMC8, CAND1, TMEM30A, CD47, RAB31, MMP25, PLD1, TYROBP, MME, ADGRE3, LILRB2, PSMA2, STXBP2, DOCK2, ATP11B, DNAJC13, CAP1, VAT1, ITGAX, TOM1, TSPAN14, VCL, CYB5R3, GCA, TIMP2, SLC11A1, PTPN6, RAB7A, FPR1, PSMB, CD14, TOLLIP, UBR4, RAB10, ANPEP, PLEKHO2, PTPRJ, TCIRG1, PSMD3, ATG7, PSMD6, MAPK1, LTA4H, MIF, UNC13D, ANXA3, PGM2, CD63, CTSB, ADGRG3, ACAA1, IRPA, IQGAP2, STK11IP, PRKCD, PSMD11, HSP90AA1, LILRA2, SYK, KPNB1, PGM1, RHOA, MLEC, MOSPD2, GUSB, DYNC1LI1, PLAUR, DIAPH1, HSPA1B, FUCA1, RAB18, ATP6V1D, SERPINA1, SIGLEC9, ARHGAP9, RAP2C, IMPDH1, VAPA, PLAC8, GLB1, PGAM1, STOM | PDCD6IP, CKAP2, CDT1, ACTR3, MYH9, APC, ARF1, TMEM250, UNC119, SNX18, RAB35, ACTR2, ROCK2, CFL1, RHOA, CHMP1B, SEPTIN9, STAMBP, CHMP6, USP8, ECT2, JTB, VPS4B, BIN3, CHMP3, ANLN, SEPTIN7, RHOB, RASA1, BRCA2, IST1, CEP55, CHMP4B, WASHC5, ROCK1, VPS4A, INCENP, CHMP7, ANXA11, SPAST | LAMP1, POLB, MAX, CUL4A, PDCD6IP, TSC2, CSRNP2, XIAP, PPP2CB, S100A8, GNB1, DPP8, PPP2R1B, STK24, NBN, DAPK2, GABARAP, NEK6, PMAIP1, ELMO2, ZPR1, PIK3CA, XKR8, PLK3, BNIP3L, C5AR1, MCL1, SHISA5, DIDO1, SGPP1, DDX3X, BRCA2, GSK3B, BBC3, FIS1, ARF6, RTN3, PDCD4, SMNDC1, HMGB1, GRK5, SLK, TP53INP1, CYCS, RNF34, NOTCH2, TNFRSF10C, TGFBR2, PSEN1, PDCD5, JUN, PRKCE, SQSTM1, PRELID1, DAPK1, ADORA2A, TNFRSF12A, BCL2L11, EIF5A, NOA1, ERO1A, HIP1, BCLAF1, CYFIP2, FXR1, SRA1, RHOB, STK17A, RBM5, TLR2, KPNA1, DEDD, MAP3K5, MAP2K4, RPS6KA3, IRF1, DNMT3A, BIRC6, TM2D1, INPP5D, RPS6KA1, RAF1, FKBP8, BCL2L1, MFF, BID, BLOC1S2, CRKL, SIAH1, PPID, RALB, CASP2, SHF, BCL3, TP53BP2, CASP8, SPI1, PDCL3, ATG4D, MX1, TAF10, GAPDH, PDK1, GSK3A, ACVR1B, LGALS1, CUL3, HTATIP2, BCL7B, MEF2D, ZMPSTE24, PPP2CA, HINT1, PRKCB, STK26, G0S2, DNAJA3, TRAF3, ATG5, AREL1, BAG1, TAOK2, ANXA6, TMBIM4, CKAP2, SGMS1, RTN4, PKN1, OPA1, FOXO3, SGPL1, ABL1, PDCD10, CTSC, PTEN, NISCH, DRAM1, BAX, TMBIM6, FEM1B, MAP3K7, S100A9, AIMP2, MSH6, PDPK1, NOTCH1, MAP2K7, PIK3R1, JTB, HIC1, PIM3, UBE2K, FADD, SH3GLB1, TNFRSF1B, TRIB3, NOD1, PRDX3, DICER1, TFDP1, PML, ATM, DDX5, HMGB2, PIM2, MYD88, PSME3, RABEP1, MTFP1, DDIT3, MELK, DEDD2, FAF1, STK4, CASP3, NF1, SUDS3, CSNK2A1, MAPK14, BIRC2, SH3KBP1, MINDY3, SIVA1, ETV6, NFKB1, HRK, TLR4, CCAR1, RYBP, ATF4, RPS27L, RNF144B, ITCH, RASSF5, STK17B, CDK5RAP3, ITGB2, ZNF622, GSN, ZNF443, MADD, ERN1, TNFRSF1A, PTK2B, STK11, KLF11, ROCK1, ZC3H8, MTCH1, UBE2D3, SHB, DFFA, DPF2, CDIP1, CIB1, TIA1, MEF2A, ZFP36L1, C1QBP, RHBDD1, DDIT4, LTBR, RB1, STK3, TIGAR, PYCARD, PAK2, MKNK2, BCL2, SELENOK, BRAT1, PIM1, PAK1, NME6, CRADD, OXR1, UBE4B, BCL2L13, SLTM, G2E3, DAPK3, CIDEB, ATAD5, PLAGL1, PPP1R15A, NFKBIA, HK2, RRAGA, SIAH2, TYROBP, TRAF7, MUL1, MTOR, SOD2, RRAGC, RIPK1, NDUFA13, IL1B, VPS35, AKTIP, TNFAIP8, PPP2R1A, RNF216, EBAG9, PPIF, SIRT1, HINT2, DDX41, PARP1, RFK, RELT, PTPN6, CD14, VDAC1, JADE1, TIAL1, TRAF1, TCIRG1, FASTK, GADD45B, KLLN, RNF130, MAPK1, TNIP2, GCLM, SORT1, BMPR2, DAP, SERPINB9, SRGN, ENDOG, TAX1BP1, TGFBR1, CDK11B, MFSD10, PKN2,EMC4, CUL5, PRKCD, GLRX2, SGK1, BUB1, HSPD1, KPNB1, TGFB1, NR3C1, PPM1F, GCLC, NUAK2, RFFL, HIPK1, CHMP3, CDK11A, MAPK3, MFN2, CXCR4, PDCD6, GADD45A, ELMO1, BCL2A1, ZFAND6, TMEM214, CUL2, CSRNP1, ARRB1, TMEM219, HRAS, HIPK3, KIF1B |
| IX | ud | KLHDC8B | SENP1, RPS6KB1, BABAM2, CASP4, TNFRSF10B |
| X | ACPP, CEACAM1, NCKAP1L, IL18RAP, MVP, DOK3, OSCAR, ABCA13, CLEC4D, CXCL8, CLEC12A, FCN1, NFASC, HK3, PRDX4 | MYH10, KIF4A | BTK, GJB6, CASP1, STPG1, GSKIP, HMOX1, IFI6, TP53, DNASE2, PHLDA3, XAF1, PAX3, SLIT2, ATP2A1, TMEM117, PIDD1, ARHGEF6, CTNNBL1, CARD6 |
| XI | NRAS, LRMP, MGAM, C6orf120 | CHMP2A | MIEN1, C6orf120, DFFB, EAF2, CDK1, FBXO10, GPR65, TAOK1, NCF1, MIR29B1 |
| XII | PRDX6, STBD1, TXNDC5, AGA, CHI3L1, SVIP, CD93, PSMB1, ITGAM, CRACR2A, MAN2B1, FCAR, GLIPR1, AGL, C1orf35, XRCC5, GPI, SRP14, CPNE3, HSPA1A, PLAU, PSMD14, CD300A, COPB1, ENPP4, HSP90AB1, BST1, ALDH3B1, TUBB, CEACAM3, DBNL, COMMD9, PSMD2, PTX3, CST3, CTSH, SPTAN1, SIRPB1, CRISPLD2, MAGT1, ITGAV, ARSB, RNASE2,NME2, ERP44, IDH1, NAPRT, ATAD3B, GLA, FGL2, LGALS3, LAMTOR3, VPS35L, PFKL, RAB3A, SIGLEC5, SLC27A2, S100A12, SNAP23, FCER1G, DYNLL1, CD53, ORMDL3, NBEAL2, FCGR3B, CFP, SURF4, ALAD, CANT1, SIGLEC14, GSDMD, MMP9, APRT, PECAM1, TMEM63A, CTSS, GALNS, CD44, FPR2, DERA, GGH, NEU1, TMC6, GOLGA7, PRCP, CXCR1, CXCL8 | DCTN3, BBS4, KIF23, RACGAP1, CNTROB, CENPA, SEPTIN6, EFHC1, AURKB, CHMP2B, CHMP4A, ZFYVE26, SEPTIN5, SNX33, ESPL1, LZTS2 | NLRP1, CHAC1, CDK5, FNIP2, TNFSF10, TNFRSF10A, MCM2, BRMS1, DNM1L, AXIN1, OGT, LY96, E2F2, ITGAM, AIFM1, PDCD7, TRADD, BNIP1, MAP3K9, MYDGF, INHBA, PUF60, HTT, STK25, TPX2, HCAR2, APP, C1D, RIPK2, HIPK2, TICAM1, BIK, RASSF7, PLSCR1, WDR92, RACK1, DELE1, XBP1, ATN1, AEN, ITGAV, THOC1, ING4, HSPA5, BRCA1, BFAR, BCL7C, BMF, FOXO1, CHEK1, XPA, ARL6IP1, BNIP3, RIPK3, USP28, SCRIB, APPL1, BRSK2, PLSCR3, DYNLL1, GHITM, BCAP31, PDCD2, POLR2G, AKT1, GSDMA, BNIP2, DNAJC10, FZD9, BCL2L12, CLPTM1L, MAP1S, PSMD10, FAIM, TNF, P2RX4, TRIM35, API5, RHOT2, PPARD, E2F1, TFPT, IFI16, TCTN3, TMEM109, CUL1, RNF41, RPS6, AKT2, CASP9, RRP1B, CCAR2, SAP30BP, GGCT, SH3RF1, ARL6IP5, TNFRSF10D, TRAF5, ZMAT3, CHI3L1, IFT57, HIP1R, GADD45G, TNFRSF19, JMY, CFLAR, DAD1, MSH2, ADARB1, PIGT, PPP1R13B, BLCAP, RMDN3, MRPL41, KMT2A, CASP8AP2, BUB1B, ERCC6, TRAIP, GRAMD4, PAK4, ERCC2, CTSH, PACS2, RHOT1, CAAP1, WWOX, TNFSF14, FZD5, TAF6, ESPL1, ERCC3, KREMEN1, MRPS30, TCHP, MAP3K10, TIMM50, DYRK2, CYP1B1, IFIT2, CD24, TRAF4, PHLPP1, ADAMTSL4, KDELR1, TNFRSF9, MYBBP1A, PHLDA1, FAM32A, APIP, ZC3H12A, DAXX, CASP6, CARD8, JAK2, C19orf12, TRAF3IP2, NUDT2, TRIM69 |
| XIII | TNFAIP6 | ANK3, TTC19 | ITPR1, RASSF6, CDKN1A, IL1A, PRKD1, SEMA3A, EGLN3, EFNA5, FAM162A, NLRC4, ATG3, RTL10 |
| XIV | CSNK2B, APAF1 | ud | APAF1, DRAM2, IER3, PRKDC |
| XV | IL18, PSMD13, GHDC, IMPDH2, TMEM173, CAMP, LYZ, FUCA2, GSTP1, CEP290, DPP7, GM2A, BPI, NCSTN, SELL, MNDA, GPR84 | CUL7, SEPTIN4, SNX9, STMN1, SEPTIN10, SPTBN1, RHOC, CIT, SPIRE1, ROPN1B | BAG3, AVEN, BAK1, ZBTB16, UXT, RPS3, MEF2C, PIK3CG, NAE1, MAGI3, PLEKHF1, IFI27L2, NOC2L, SH3RF3, CIDEC, ITGB3BP, PDK2, SMAD3, NCSTN, XKR6, APBB1, SEPTIN4, SAV1, RNF152, MLH1, CAPN10, TNFSF12, EI24, FHIT, NME3, CIT, TOP2A, CASP7, MAP2K6, AHR |

ud – undetectable
