# Supplementary material for: Changes Within H3K4me3-Marked Histone Reveal Molecular Background of Neutrophil Functional Plasticity
Source: Front Immunol. 2022 Jun 10;13:906311. doi: 10.3389/fimmu.2022.906311 (PMC9229595; doi:10.3389/fimmu.2022.906311)
Supplement: Supplementary Table 5 — The list of all tested mRNA for genes associated with activation of neutrophils via NF-κB. Data are presented as the relative fold differences to n.s. neutrophils. ‘H3K4me3 TSS regions’ column presents genes that are positioned by H3K4me3. Total count readings ≥10, within the first intron, were set as the detection limit. [file Table_5.docx]

**Supplementary** **Table S5**. The list of all tested mRNA for genes associated with activation of neutrophils *via* NF-κB. Data are presented as the relative fold differences to n.s. neutrophils. ‘H3K4me3 TSS regions’ column presents genes which are positioned by H3K4me3. Total count readings ≥10, within first intron, was set as detection limit.

| Gene | | RefSeq ID | Relative fold differences | | | H3K4me3  TSS regions |
| --- | --- | --- | --- | --- | --- | --- |
|  |  |  | **LPS *vs*. n.s.** | **TNF-α  *vs.* n.s.** | **IL-10 *vs.* n.s.** |  |
| AGT | Adrenomedullin | NM_001124 | 1,613 | 1,415 | 1,311 | TNF-α |
| ADM | Angiotensinogen (serpin peptidase inhibitor, clade A, member 8) | NM_000029 | 3,915 ^#^ | 2,058 ^#^ | 1,304 | n.s. IL-10, LPS |
| AKT1 | V-akt murine thymoma viral oncogene homolog 1 | NM_005163 | -2,120 ^#^ | -2,517 ^#^ | 1,176 | n.s., IL-10, LPS |
| ATF1 | Activating transcription factor 1 | NM_005171 | -1,184 | -1,626 ^#^ | 1,216 | IL-10, TNF-α |
| ALDH3A2 | Aldehyde dehydrogenase 3 family, member A2 | NM_000382 | -1,228 | -2,425 | -1,609 | n.s., IL-10, LPS |
| BCL10 | BCL2-related protein A1 | NM_004049 | 1,293 | 1,182 | -1,082 | n.s., IL-10, LPS |
| BCL2L1 | BCL2-like 1 | NM_138578 | 5,477 ^#^ | 7,840 ^#^ | -3,880 ^#^ | n.s., IL-10, LPS |
| BCL3 | B-cell CLL/lymphoma 3 | NM_005178 | 1,981 ^#^ | 2,573 ^#^ | 1,056 | n.s., IL-10, LPS |
| BIRC2 | Baculoviral IAP repeat containing 2 | NM_001166 | -2,017 | -1,658 | 1,663 | n.s., IL-10, LPS |
| BIRC3 | Baculoviral IAP repeat containing 3 | NM_001165 | 2,128 | 5,072 ^#^ | 1,196 | IL-10, LPS |
| C3 | Complement component 3 | NM_000064 | 6,957 ^#^ | 4,681 ^#^ | -1,119 | not detectable |
| CARD11 | Caspase recruitment domain family, member 11 | NM_032415 | -2,113 | -3,792 ^#^ | 1,113 | not detectable |
| CASP1 | Caspase 1, apoptosis-related cysteine peptidase | NM_033292 | 1,311 | 1,195 | 2,098 | n.s. IL-10, LPS |
| CASP8 | Caspase 8, apoptosis-related cysteine peptidase | NM_001228 | 3,413 ^#^ | 4,183 ^#^ | 1,269 | n.s., IL-10, LPS |
| Eotoxin (CCL11) | Chemokine (C-C motif) ligand 11 | NM_002986 | -1,810 | -1,068 | -3,109^#^ | not detectable |
| MCP1  (CCL2) | Chemokine (C-C motif) ligand 2 | NM_002982 | 2,426 ^#^ | 3,421 ^#^ | 1,347 | not detectable |
| MDC  (CCL22) | Chemokine (C-C motif) ligand 22 | NM_002990 | -1,558 | -1,466 | -1,987 | IL-10 |
| CCND1 | Cyclin D1 | NM_053056 | -1,004 | 1,672 | -1,634 | n.s., IL-10, LPS |
| CCR5 | Chemokine (C-C motif) receptor 5 | NM_000579 | 1,890 ^#^ | 2,501 ^#^ | -1,340 | not detectable |
| CD27 | CD27 molecule | NM_001242 | 1,268 | 7,478 ^#^ | 1,281 | not detectable |
| CD40 | CD40 molecule, TNF receptor superfamily member 5 | NM_001250 | 3,911 ^#^ | 3,080 ^#^ | -1,623 | not detectable |
| CD69 | CD69 molecule | NM_001781 | 3,694 ^#^ | 3,091 ^#^ | -1,024 | not detectable |
| CD80 | CD80 molecule | NM_005191 | 2,578 ^#^ | 3,482 ^#^ | -1,634 | not detectable |
| CD83 | CD83 molecule | NM_004233 | 4,435 ^#^ | 4,971 ^#^ | -1,338 | IL-10, LPS |
| CDKN1A | Cyclin-dependent kinase inhibitor 1A (p21, Cip1) | NM_000389 | 3,150 ^#^ | 4,860 ^#^ | 1,197 | IL-10, LPS |
| CFB | Complement factor B | NM_001710 | 1,085 | 1,522 | -1,819 ^#^ | not detectable |
| CFLAR | CASP8 and FADD-like apoptosis regulator | NM_003879 | -1,898 | -3,140 ^#^ | 1,281 | n.s., IL-10, LPS |
| CHUK  (IKK1) | Conserved helix-loop-helix ubiquitous kinase  (Inhibitor of nuclear factor kappa-B kinase subunit alpha) | NM_001278 | 2,250 ^#^ | 3,419 ^#^ | -2,334 ^#^ | n.s., IL-10, LPS |
| CSF1 | Colony stimulating factor 1 (macrophage) | NM_000757 | 2,380 ^#^ | 5,901 ^#^ | -1,150 | n.s., IL-10, LPS |
| CSF2 | Colony stimulating factor 2 (granulocyte-macrophage) | NM_000758 | 1,724 | 6,570 ^#^ | 1,699 | not detectable |
| CSF2RB | Colony stimulating factor 2 receptor | NM_000395 | 1,125 | -1,408 ^#^ | 1,370 | n.s., IL-10, LPS |
| CSF3 | Colony stimulating factor 3 | NM_000759 | 2,560 ^#^ | 6,724 ^#^ | 1,971 | not detectable |
| EGFR | Epidermal growth factor receptor | NM_005228 | 1,153 | 1,357 | 1,290 | not detectable |
| EGR1 | Early growth response 1 | NM_001964 | -4,148 ^#^ | -1,725 | 1,894 | n.s., IL-10, LPS |
| EGR2 | Early growth response 2 | NM_000399 | 4,750 ^#^ | 4,171 ^#^ | -1,028 | n.s., IL-10, LPS |
| ELK1 | ELK1, member of ETS oncogene family | NM_005229 | 1,717 | 1,526 | 1,159 | n.s., IL-10, LPS |
| F3 | Coagulation factor III (thromboplastin, tissue factor) | NM_001993 | 4,808 ^#^ | 6,655 ^#^ | 1,100 | IL-10, LPS |
| F8 | Coagulation factor VIII, procoagulant component | NM_000132 | 1,433 | 2,209 ^#^ | 1,057 | not detectable |
| F2R | Coagulation factor II (thrombin) receptor | NM_001992 | -1,038 | -1,357 | 1,288 | IL-10 |
| FADD | Fas (TNFRSF6)-associated via death domain | NM_003824 | 1,459 | 1,186 | 1,086 | n.s., IL-10, LPS |
| FAS | Fas (TNF receptor superfamily, member 6) | NM_000043 | 1,922 | 1,084 | -1,289 | n.s., IL-10, LPS |
| FASLG | Fas ligand (TNF superfamily, member 6) | NM_000639 | 1,165 | 1,492 | 3,246 ^#^ | not detectable |
| FOS | FBJ murine osteosarcoma viral oncogene homolog | NM_005252 | 2,394 ^#^ | 2,208 ^#^ | 1,152 | n.s., IL-10, LPS |
| GADD45B | Growth arrest and DNA-damage-inducible, beta | NM_015675 | 3,179 ^#^ | 2,860 ^#^ | -1,486 | n.s., IL-10, LPS, TNF-α |
| HMOX1 | Heme oxygenase (decycling) 1 | NM_002133 | 2,636 ^#^ | 11,926 ^#^ | 1,301 | IL-10 |
| ICAM1 | Intercellular adhesion molecule 1 | NM_000201 | 3,189 ^#^ | 8,396 ^#^ | -1,093 | IL-10, LPS |
| IFNA1 | Interferon, alpha 1 | NM_024013 | 1,404 | 1,013 | -1,071 | not detectable |
| IFNG | Interferon, gamma | NM_000619 | 1,888 | 1.576 | 1,785 | not detectable |
| IFNB1 | Interferon, beta 1, fibroblast | NM_002176 | 2,353 ^#^ | 1,295 | -1,338 | not detectable |
| IKBKB  (IKK2) | Inhibitor of kappa light polypeptide gene enhancer in B-cells, kinase beta | NM_001556 | 2,613 ^#^ | 3,032 ^#^ | 1,528 | IL-10, LPS |
| IKBKE | Inhibitor of kappa light polypeptide gene enhancer in B-cells, kinase epsilon | NM_014002 | 3,500 ^#^ | 3,321 ^#^ | 1,320 | IL-10 |
| IKBKG  (NEMO) | Inhibitor of kappa light polypeptide gene enhancer in B-cells, kinase gamma (NF-kappa-B essential modulator) | NM_003639 | 3,037 ^#^ | 1,271 | 1,084 | n.s., IL-10, LPS |
| IL10 | Interleukin 10 | NM_000572 | 1,633 | 1,320 | 3,888 ^#^ | IL-10 |
| IL1A | Interleukin 1, alpha | NM_000575 | 4,516 ^#^ | 7,024 ^#^ | 1,485 | not detectable |
| IL1B | Interleukin 1, beta | NM_000576 | 4,092 ^#^ | 3,496 ^#^ | 1,212 | n.s. IL-10, LPS |
| IL1R1 | Interleukin 1 receptor, type I | NM_000877 | 1,762 | 3,543 ^#^ | 1,375 | not detectable |
| IL1R2 | Interleukin 1 receptor, type II | NM_004633 | 1,761 | 1,737 | -1,094 | n.s., IL-10, LPS |
| IL1RN | Interleukin 1 receptor antagonist | NM_000577 | 5,259 ^#^ | 6,057 ^#^ | -1,604 | not detectable |
| IL12B | Interleukin 12B (natural killer cell stimulatory factor 2, cytotoxic lymphocyte maturation factor 2, p40) | NM_002187 | 3,529 ^#^ | 1,626 | 1,320 | not detectable |
| IL15 | Interleukin 15 | NM_000585 | -1,006 | -1,687 | -1,627 | IL-10 |
| IL2 | Interleukin 2 | NM_000586 | -1,421 | -1,135 | 1,020 | not detectable |
| IL2RA | Interleukin 2 receptor, alpha | NM_000417 | 1,427 | 3,643 ^#^ | -1,362 | not detectable |
| IL4 | Interleukin 4 | NM_000589 | -2,017 | -1,438 | -1,558 | not detectable |
| IL6 | Interleukin 6 (interferon, beta 2) | NM_000600 | 6,948 ^#^ | 4,053 ^#^ | 4,819 ^#^ | not detectable |
| Gro-α  (CXCL1) | Chemokine (C-X-C motif) ligand 1 (melanoma growth stimulating activity, alpha) | NM_001511 | 1,962 | -1,198 ^#^ | -1,100 | n.s., IL-10, LPS |
| IP-10  (CXCL10) | Chemokine (C-X-C motif) ligand 10 | NM_001565 | 2,998 ^#^ | 1,196 | -1,643 | not detectable |
| Gro-β  (CXCL2) | Chemokine (C-X-C motif) ligand 2 | NM_002089 | 2,494 ^#^ | 2,443 ^#^ | -1,133 | n.s., IL-10, LPS |
| IL-8  (CXCL8) | Interleukin 8 | NM_000584 | 5,855 ^#^ | 2,142 ^#^ | 2,668 ^#^ | n.s., IL-10, LPS |
| CXCL9  (MIG) | Chemokine (C-X-C motif) ligand 9 | NM_002416 | -1,159 | 1,129 | -1,290 | not detectable |
| IRAK1 | Interleukin-1 receptor-associated kinase 1 | NM_001569 | 3,058 ^#^ | 2,237 ^#^ | -1,114 | n.s., IL-10, LPS |
| IRAK2 | Interleukin-1 receptor-associated kinase 2 | NM_001570 | 3,992 ^#^ | 2,147 ^#^ | 3,173^#^ | n.s., IL-10, LPS |
| INS | Insulin | NM_000207 | 1,189 | 1,360 | -1,477 | not detectable |
| IRF1 | Interferon regulatory factor 1 | NM_002198 | 3,982 ^#^ | 5,797 ^#^ | 1,492 | n.s., IL-10, LPS, TNF-α |
| JUN | Jun proto-oncogene | NM_002228 | 1,381 | 1,968 | 2,299 | n.s., IL-10, LPS |
| LTA | Lymphotoxin alpha (TNF superfamily, member 1) | NM_000595 | 2,749 ^#^ | 1,069 | 1,368 | not detectable |
| LTB | Lymphotoxin beta (TNF superfamily, member 3) | NM_002341 | 2,495 ^#^ | 1,399 | 1,386 | n.s., IL-10, LPS |
| LTBR | Lymphotoxin beta receptor (TNFR superfamily, member 3) | NM_002342 | -1,244 | -1,868 | 1,804 | n.s., IL-10, LPS |
| MALT1 | Mucosa associated lymphoid tissue lymphoma translocation gene 1 | NM_173844 | -1,231 | -1,059 | 1,227 | not detectable |
| MAP3K1 | Mitogen-activated protein kinase 1 | NM_005921 | 2,800 ^#^ | -2,272 ^#^ | 3,094 ^#^ | n.s., IL-10, LPS |
| MAP2K6 | Mitogen-activated protein kinase 6 | NM_002758 | -1,758 ^#^ | -2,372 ^#^ | 1,072 | not detectable |
| MMP9 | Matrix metallopeptidase 9 (gelatinase B, 92kDa gelatinase, 92kDa type IV collagenase) | NM_004994 | 1,009 | -1,025 | 1,050 | IL-10, LPS |
| MYC | V-myc myelocytomatosis viral oncogene homolog (avian) | NM_002467 | 2,349 ^#^ | 1,533 | 1,166 | not detectable |
| MYD88 | Myeloid differentiation primary response gene (88) | NM_002468 | 2,043 ^#^ | 1,913 ^#^ | 1,392 | n.s., IL-10, LPS, TNF-α |
| NCOA3 | Nuclear receptor coactivator 3 | NM_181659 | -2,204 ^#^ | -3,033 ^#^ | -1,277 | n.s., IL-10, LPS, TNF-α |
| NFKB1 | Nuclear factor of kappa light polypeptide gene enhancer in B-cells 1 | NM_003998 | 3,149 ^#^ | 10,068 ^#^ | 1,221 | n.s. ,IL-10, LPS, TNF-α |
| NFKB2 | Nuclear factor of kappa light polypeptide gene enhancer in B-cells 2 (p49/p100) | NM_002502 | 3,299 ^#^ | 7,890 ^#^ | 2,290 ^#^ | n.s., IL-10, LPS |
| NFKBIA | Nuclear factor of kappa light polypeptide gene enhancer in B-cells inhibitor, alpha | NM_020529 | 3,889 ^#^ | 8,258 ^#^ | 1,016 | n.s., IL-10, LPS |
| NFKBIB | Nuclear factor of kappa light polypeptide gene enhancer in B-cells inhibitor, beta | NM_002503 | 4,593 ^#^ | 7,428^#^ | -1,236 | n.s., IL-10, LPS |
| NFKBIE | Nuclear factor of kappa light polypeptide gene enhancer in B-cells inhibitor, epsilon | NM_004556 | -1,788 | -2,196 ^#^ | 1,146 | IL-10, LPS |
| NOD1 | Nucleotide-binding oligomerization domain containing 1 | NM_006092 | -1,409 | -1,777 ^#^ | 1,190 | n.s., IL-10, LPS |
| NQO1 | NAD(P)H dehydrogenase, quinone 1 | NM_000903 | 1,114 | 1,303 | 1,085 | IL-10 |
| NR4A2 | Nuclear receptor subfamily 4, group A, member 2 | NM_006186 | 1,831 | 2,554 ^#^ | -1,151 | n.s., IL-10, LPS |
| PDGFB | Platelet-derived growth factor beta polypeptide | NM_002608 | -1,066 | 1,728 | -1,285 | not detectable |
| PLAU | Plasminogen activator, urokinase | NM_002658 | 4,962 ^#^ | 3,007 ^#^ | 1,055 | n.s., IL-10, LPS |
| PTGS2 | Prostaglandin-endoperoxide synthase 2 (prostaglandin G/H synthase and cyclooxygenase) | NM_000963 | 1,502 | 1,162 | -1,048 | n.s., IL-10, LPS |
| PSIP1 | PC4 and SFRS1 interacting protein 1 | NM_021144 | 3,407 ^#^ | 2,579 ^#^ | 1,182 | n.s,. IL-10, LPS, TNF-α |
| RAF1 | V-raf-1 murine leukemia viral oncogene homolog 1 | NM_002880 | 3,332 ^#^ | 2,233 ^#^ | 1,258 | n.s., IL-10, LPS, TNF-α |
| REL | V-rel reticuloendotheliosis viral oncogene homolog | NM_002908 | 3,121 ^#^ | 2,689 ^#^ | 1,567 | n.s. IL-10, LPS, TNF-α |
| RELA | V-rel reticuloendotheliosis viral oncogene homolog A (avian) | NM_021975 | 7,346^#^ | 10,884 ^#^ | 1,041 | n.s., IL-10, LPS |
| RELB | V-rel reticuloendotheliosis viral oncogene homolog B | NM_006509 | 1,162 | 1,462 | 5,55 ^#^ | n.s., IL-10, LPS |
| RHOA | Ras homolog gene family, member A | NM_001664 | 1,174 | -1,380 ^#^ | 1,707 | n.s., IL-10, LPS |
| RIPK1 | Receptor (TNFRSF)-interacting serine-threonine kinase 1 | NM_003804 | 1,288 | 1,642 | 2,480 | n.s., IL-10, LPS, TNF-α |
| SELE | Selectin E | NM_000450 | -1,857 | -1,169 | -2,688 ^#^ | not detectable |
| SELP | Selectin P (granule membrane protein 140kDa, antigen CD62) | NM_003005 | -2,384 ^#^ | -1,167 | 1,169 | not detectable |
| SNAP25 | Synaptosomal-associated protein, 25kDa | NM_003081 | -2,019 ^#^ | 1,019 | -1,707 | not detectable |
| SOD2 | Superoxide dismutase 2, mitochondrial | NM_000636 | 3,840 ^#^ | 4,667 ^#^ | -1,212 | n.s., IL-10, LPS |
| STAT1 | Signal transducer and activator of transcription 1, 91kDa | NM_007315 | -1,025 | -1,374 ^#^ | 1,053 | n.s., IL-10, LPS |
| STAT3 | Signal transducer and activator of transcription 3 (acute-phase response factor) | NM_003150 | -1,098 | -1,256 | 1,085 | n.s., IL-10, LPS |
| STAT5B | Signal transducer and activator of transcription 5B | NM_012448 | 1,141 | -1,023 | 1,531 | n.s., IL-10, LPS |
| TBK1 | TANK-binding kinase 1 | NM_013254 | 2,155 ^#^ | 2,383 ^#^ | 1,025 | n.s., IL-10, LPS |
| TICAM1 | Toll-like receptor adaptor molecule 1 | NM_182919 | -1,748 | -2,388 ^#^ | 1,211 | IL-10, LPS |
| TICAM2 | Toll-like receptor adaptor molecule 2 | NM_021649 | -1,967 | -3,638 ^#^ | 1,279 | n.s., IL-10, LPS |
| TIMP1 | TIMP metallopeptidase inhibitor 1 | NM_003254 | 1,596 | 2,116 ^#^ | 1,238 | not detectable |
| TLR1 | Toll-like receptor 1 | NM_003263 | 1,050 | 1,169 | -1,220 | n.s. IL-10, LPS |
| TLR2 | Toll-like receptor 2 | NM_003264 | 1,364 | 1,762 ^#^ | 1,038 | n.s., IL-10, LPS |
| TLR3 | Toll-like receptor 3 | NM_003265 | -2,097 | -3,652 ^#^ | 1,447 | not detectable |
| TLR4 | Toll-like receptor 4 | NM_138554 | 1,183 | 1,165 | 1,871 | n.s., IL-10, LPS |
| TLR6 | Toll-like receptor 6 | NM_006068 | -3,536 ^#^ | -2,483 ^#^ | 1,029 | n.s., IL-10, LPS |
| TLR9 | Toll-like receptor 9 | NM_017442 | -1,008 | -1,032 | 2,919 | not detectable |
| TNF | Tumor necrosis factor | NM_000594 | 10,397 ^#^ | 2,726 ^#^ | -2,167 ^#^ | IL-10, LPS |
| TNFAIP3 | Tumor necrosis factor receptor superfamily, member 1B | NM_001066 | 4,080 ^#^ | 3,766 ^#^ | -1,104 | n.s., IL-10, LPS |
| TNFRSF10A | Tumor necrosis factor (ligand) superfamily, member 10 | NM_003810 | 3,384 ^#^ | 4,271 ^#^ | 1,122 | IL-10 |
| TNFRSF10B | Tumor necrosis factor receptor superfamily, member 10b | NM_003842 | 1,570 | 1,415 | 1,954 | n.s., IL-10, LPS |
| TNFRSF1A | Tumor necrosis factor receptor superfamily, member 1A | NM_001065 | 1,466 | 1,258 | 1,609 | n.s., IL-10, LPS |
| TNFRSF1B | Tumor necrosis factor receptor superfamily, member 1B | NM_001066 | 2,576 | 2,418 | 1,739 | n.s., IL-10, LPS |
| TNFSF10 | Tumor necrosis factor (ligand) superfamily, member 10 | NM_003810 | -6,532 ^#^ | -6,664^#^ | -1,070 | n.s., IL-10, LPS |
| TNFSF14 | Tumor necrosis factor (ligand) superfamily, member 14 | NM_003807 | -1,056 | -1,167 | 1,546 | IL-10, LPS |
| TP53 | Tumor protein p53 | NM_000546 | 1,068 | 2,287 ^#^ | 1,154 | IL-10, LPS |
| TRADD | TNFRSF1A-associated via death domain | NM_003789 | -4,216 ^#^ | -3,352 ^#^ | 1,157 | IL-10, LPS |
| TRAF2 | TNF receptor-associated factor 2 | NM_021138 | -3,210^#^ | -7,688 ^#^ | 3,770 ^#^ | IL-10 |
| TRAF3 | TNF receptor-associated factor 3 | NM_003300 | -2,375 ^#^ | -3,096^#^ | 4,261 ^#^ | IL-10, LPS |
| TRAF6 | TNF receptor-associated factor 6 | NM_004620 | 7,568 ^#^ | 3,257 ^#^ | 1,358 | IL-10, LPS |
| VCAM1 | Vascular cell adhesion molecule 1 | NM_001078 | -2,241 | 1,276 | -2,380 ^#^ | not detectable |
| XIAP | X-linked inhibitor of apoptosis | NM_001167 | -1,032 | -1,062 | -3,042 ^#^ | n.s., IL-10, LPS |

#- statistically significant differences to n.s. neutrophils; not detectable – the value of total counts <10.
